# Characterization of the exopolymer-producing *Pseudoalteromonas* sp. S8-8 from Antarctic sediment

**DOI:** 10.1007/s00253-022-12180-x

**Published:** 2022-09-26

**Authors:** Carmen Rizzo, Elena Perrin, Annarita Poli, Ilaria Finore, Renato Fani, Angelina Lo Giudice

**Affiliations:** 1 Marine Biotechnology Department, Stazione Zoologica “Anton Dohrn”, Sicily Marine Centre, Villa Pace, Messina, Italy; 2 Department of Biology, University of Florence, Florence, Italy; 3 Institute of Biomolecular Chemistry, National Research Council (ICB-CNR), Pozzuoli, NA) Italy; 4 Institute of Polar Sciences, National Research Council (CNR-ISP), Spianata San Raineri 86, 98122 Messina, Italy; 5 Italian Collection of Antarctic Bacteria, National Antarctic Museum (CIBAN-MNA), Section of Messina, Messina, Italy

**Keywords:** Cold adapted, EPS, Cryoprotection, Genome, Biotechnology

## Abstract

**Abstract:**

A synergistic approach using cultivation methods, chemical, and bioinformatic analyses was applied to explore the potential of *Pseudoalteromonas* sp. S8-8 in the production of extracellular polymeric substances (EPSs) and the possible physiological traits related to heavy metal and/or antibiotic resistance. The effects of different parameters (carbon source, carbon source concentration, temperature, pH and NaCl supplement) were tested to ensure the optimization of growth conditions for EPS production by the strain S8-8. The highest yield of EPS was obtained during growth in culture medium supplemented with glucose (final concentration 2%) and NaCl (final concentration 3%), at 15 °C and pH 7. The EPS was mainly composed of carbohydrates (35%), followed by proteins and uronic acids (2.5 and 2.77%, respectively) and showed a monosaccharidic composition of glucose: mannose: galactosamine: galactose in the relative molar proportions of 1:0.7:0.5:0.4, as showed by the HPAE-PAD analysis. The detection of specific molecular groups (sulfates and uronic acid content) supported the interesting properties of EPSs, i.e. the emulsifying and cryoprotective action, heavy metal chelation, with interesting implication in bioremediation and biomedical fields. The analysis of the genome allowed to identify a cluster of genes involved in cellulose biosynthesis, and two additional gene clusters putatively involved in EPS biosynthesis.

**Key points:**

• *A cold-adapted Pseudoalteromonas strain was investigated for EPS production.*

• *The EPS showed emulsifying, cryoprotective, and heavy metal chelation functions.*

• *Three gene clusters putatively involved in EPS biosynthesis were evidenced by genomic insights.*

**Supplementary Information:**

The online version contains supplementary material available at 10.1007/s00253-022-12180-x.

## Introduction

The search for new molecules of natural origin, which could be used as alternatives to chemically synthesized compounds, is one of the most urgent goals in the modern biotechnology. The production of biomolecules with functional traits of ecological and biotechnological interest by microorganisms living in extremely cold environments is a subject that is as fascinating as it is still relatively little explored. Extracellular polymeric substances are among the most interesting compounds, with a wide spectrum of ecological functions, that can be transferred in industrial processes and medical field applications (Poli et al. 2017; Lo Giudice et al. 2020). The development of new analytical approaches and the coupling of cultivation techniques to genomics have represented the driving force in recent times to push research in these areas. Many implications can emerge from the deeper knowledge of the taxonomic groups of relevance for biotechnological purposes, from the chemical structure of bioproducts to the optimal conditions for the biosynthetic processes, in addition to reveal important details on the strategies adopted by these organisms for the survival in adverse conditions. Extracellular polymeric substances (EPSs) are molecules of polysaccharidic composition naturally produced by microorganisms in different forms, as powerful defensive agents able to implement the survival in harsh environmental conditions. Additionally, many studies highlighted the ecological role of EPSs on large scale, giving them a specific function in the total organic balance in aquatic environments (Krembs et al. 2002). The new challenges, which research in biotechnology must face in an intense technological development era, require a synergistic approach involving different methodologies and skills. It engages ecological competences, together with increasingly sophisticated and avant-garde methods of extraction and purification, aimed at elucidating the EPS chemical structures and, in turn, their structure-related predicted functions.

Ecological and chemical skills are the current cornerstones in the field of bioprospecting, in which some issues are still underexplored. Knowledge of microbial biodiversity allows us to detect new sources with higher potential for the isolation of producers with rare functionality, that is why extreme environments have recently emerged as a promising pool. As reviewed by Lo Giudice et al. (2020), EPSs have been found in the Antarctic areas as component of dissolved organic carbon, particulate material, biofilms, and sea ice microbial communities (Mancuso Nichols et al. 2005a, b) with pivotal role in the survival strategies. The cryosphere includes a set of highly diversified habitats, with multiple adverse conditions that microorganisms must cope with by implementing increasingly specific adaptation strategies not found in other environments. Some recent contributions have made it possible to explore the potential of several Antarctic bacteria of different origins (i.e., seawater and benthic invertebrates), elucidating the most influencing parameters on the production of EPS and their peculiar chemical characteristics. EPSs were shown to play a key role as cryoprotective agents and emulsifiers; moreover, an interesting potential in the ability to chelate heavy metals was alto revealed (Caruso et al. 2018a,b, 2019).

Several taxonomic groups have been reported as EPS producers from both Arctic and Antarctic environments, e.g., *Pseudoalteromonas*, *Marinobacter*, *Polaribacter*, *Shewanella*, *Colwellia*, *Winogradskyella*, and *Pseudomonas* (Casillo et al. 2018; Caruso et al. 2019). Representatives of the genus *Pseudoalteromonas* are the most studied in the field, as proof of concept that the few EPSs chemically characterized are produced by its affiliates (Corsaro et al. 2004; Kim and Yim 2007; Mancuso Nichols et al. 2004, 2005a), considered particularly promising for biotechnological purposes. Despite this, some aspects still deserve to be explored, such as the poorly assessed heavy metal chelation and protection against freezing processes (Loaëc et al. 1998). Moreover, the genetic traits of these strains have been not yet related to their physiological functions or to the production of EPSs. The genome mining approach allows to identify gene sequences involved in the metabolic and physiological processes of the organisms, by revealing potentials that are not always expressed, but present. The comparison with other congeners also provides useful information on the evolutionary level or to accurately detect the onset of gene mutations responsible for the development of new functions.

*Pseudoalteromonas* sp. S8-8 was isolated from Antarctic shallow sediment (15-m depth) in the Road Bay (Terra Nova Bay, Ross Sea; coordinates: 74°41′80.3″S–164°307′80.3″E) (Lo Giudice et al. 2013). The genome sequence of this strain was previously obtained (Bosi et al. 2015) and included in a genome-scale phylogenetic analysis of the *Pseudoalteromonas* genus that allowed to place this strain in the clade of the non-pigmented *Pseudoalteromonas haloplanktis*-like group (Bosi et al. 2015, 2017). Moreover, the *Pseudoalteromonas* pangenome has been reconstructed and some interesting features of the *Pseudoalteromonas* representatives, such as the production of secondary metabolites, the adaptation to cold temperatures, and the resistance to abiotic compounds have been identified (Bosi et al. 2017).

This study was aimed at elucidating the potential of *Pseudoalteromonas* sp. S8-8 in EPS production with implication of biotechnological interest and to assess putative correlations with genetic traits of its genome.

## Material and methods

### Bacterial strain

*Pseudoalteromonas* sp. S8-8 was selected for further analyses as it showed an evident mucous phenotype during growth in liquid culture (Marine Broth, MB; Difco) and solid medium (Marine Agar, MA; Difco) supplemented with glucose (0.6%, w/v) (Lo Giudice 2006). The strain was identified by the 16S rRNA gene sequencing and analysis (Michaud et al. 2004) and the *Pseudoalteromonas* sp. S8-8 genome sequence has been deposited in the NCBI GenBank database (NCBI Reference Sequence: NZ_AUTR01000035.1) (Bosi et al. 2015). *Pseudoalteromonas* sp. S8-8 (museal code: MNA-CIBAN-0096) belongs to the Italian Collection of Antarctic Bacteria of the National Antarctic Museum (CIBAN-MNA) kept at the University of Messina (Italy).

### Phenotypic characterization of Pseudoalteromonas sp. S8-8

The phenotypic characterization of *Pseudoalteromonas* sp. S8-8 was performed as reported by Lo Giudice et al. (2012). The morphology and pigmentation of colonies were determined after growth on MA plates at 4 °C, and the presence of flagella was verified by using the Bacto Flagella Stain (Difco). Gram reaction, motility, and endospore presence were also analyzed. Optimal temperature and pH ranges were detected by growing the strain in MB under different experimental conditions (temperature, 4, 15, 20, 25, 30, and 37 °C; pH, 4, 5, 6, 7, 8, and 9). Growth in presence of 0–5% (w/v) NaCl concentration was tested on Nutrient Agar (NA). The presence of oxidase and catalase activities were determined. Hydrolysis of chitin, agar, and starch and lipolytic activity were assayed. API tests (BioMerieux), including API 20E and API 20NE galleries, were used as additional biochemical and enzymatic tests. Susceptibility to chloramphenicol (30 μg), tetracycline (30 μg), nalidixic acid (30 μg), penicillin G (10 μg), polymyxin B (30 μg), tobramycin (10 μg), and vibriostatic agent O/129 (10 μg) was assayed by using the standard disk diffusion method and expressed in terms of sensitivity or total resistance. For tests carried out on solid and liquid media, cultures were incubated at 4 °C for 21 days. All analyses were performed at least twice to confirm results.

### EPS production and extraction

#### Optimization of the EPS production

Optimal growth conditions for EPS production by the strain S8-8 were assayed by a step-by-step experiment, as previously reported (Caruso et al. 2018a, b, 2019). Aliquots of bacterial pre-culture (10%, v/v) were used to inoculate 300 mL of a minimal medium (Caruso et al. 2018a,b, 2019). The bacterial cultures were incubated at 4 and/or 15 °C and spectrophotometrically monitored to measure bacterial growth (UV-mini-1240, Shimadzu) at λ 600 nm (OD_600_) at regular intervals. The EPS production was concomitantly determined through the phenol–sulfuric acid method (Dubois et al. 1956). In a first phase, two different carbon sources have been tested at temperature of 4 and 15 °C (glucose and sucrose, final concentration 0.6%, w/v), to then evaluate carbon source concentration (range 0.6–2%, w/v), pH (range 6–8), and salinity (range NaCl 1–5%, w/v) effects.

One-way ANOVA and the Tukey test were inferred to detect significant influence of external parameters in the EPS production by *Pseudoalteromonas* sp. S8-8 during growth under different conditions, thus assessing any significant difference between variables (MiniTab software, version 16.0; significance level 0.05).

#### EPS extraction from the culture medium

EPSs were extracted as previously reported in Caruso et al. (2018a, b, 2019) by merging two different methods (Joulak et al. 2019, 2020; Rinker and Kelly 1996). *Cell-free* broth culture obtained by centrifugation (8000 × *g* for 10 min at 4 °C) was treated with an equal volume of cold ethanol and stored overnight at − 20 °C. The pellet precipitate thus obtained was separated from the liquid phase by centrifugation at 10,000 × *g* for 30 min and dissolved in hot water. All the steps have been carried out twice. Raw extracts were freeze-dried and weighted after dialysis performed against tap water (48 h) and distilled water (24 h).

#### EPS characterization

A set of colorimetric assays have been performed as preliminary chemical characterization of the EPSs extracted from S8-8 cultures. In detail, carbohydrate, protein, and uronic acid contents have been determined by using respectively the Dubois method (1956), the Coomassie Brilliant Blue (Bradford 1976) assay, and the method by Blumenkrantz and Asboe-Hansen (1973), modified by Filisetti-Cozzi and Carpita (1991).

Sugar composition was furtherly investigated after EPS hydrolysis with a 2 M trifluoroacetic acid solution at 120 °C for 2 h, by thin-layer chromatography and high-pressure anion-exchange pulsed amperometric detection (HPAE-PAD) with sugar standards for identification and calibration curves (Joulak et al. 2019, 2020; Finore et al. 2016).

Fourier transform infrared spectroscopy (FT-IR) was used to detect and characterize the major structural groups, in particular the C = O bonds and O–H bonds in the range of 4000–400 cm^−1^, and sulfate content in the range 1250–1050 cm^−1^ (Lijour et al. 1994).

#### Biotechnological potential of the EPSs

The EPSs produced by S8-8 were screened for their emulsifying activity, cryoprotective action, and heavy metal tolerance enhancement, as previously described by (Caruso et al. 2018b, 2019).

The stable emulsion index (E_24_) was determined as a measure of emulsifying activity, by vigorously shaking an equal volume of lyophilized EPS solution (in distilled water; 0.5%, w/v) and standard hydrocarbons (hexane, Baker; octane, hexadecane, and tetradecane, Sigma). The emulsion was measured after 24 h (Satpute et al. 2008). Tween 80 (Biomedicals) and Triton X-100 (Sigma) were used as surfactant positive controls.

The cryoprotective effect of EPS was evaluated by submitting to four freeze–thaw cycles bacterial biomass (1 mL), obtained by harvesting (10,000 × *g* for 20’, 4 °C) liquid cultures in presence and absence of EPSs. Cell viability was evaluated spectrophotometrically at the end of each thawing both to evaluate the cryoprotective effect (Li et al. 2006).

Heavy metal tolerance of *Pseudoalteromonas* sp. S8-8 was investigated (i.e., cadmium, mercury, zinc, and iron; range 10–10,000 ppm) both in presence and absence of sugar (0.6%, w/v) by the plate diffusion method (Selvin et al. 2009). The method was applied as reported by Mangano et al. (2014), bycreating a central well on solidified medium sealed with soft agar (0.8% agar, w/v) in which an aliquot of metal salt solution (0.5 mL; in sterile phosphate buffer saline, PBS) was added. The strain was streaked in radial streaks and let to grow at 4 °C for 21 days. Sterile PBS was used as a negative control. Bacterial tolerance was expressed as the ratio between the length of growth in mm vs the length of total inoculated streak. Tolerance ranges were classified in complete (100% of growth), high (≥ 50–99% of growth), low (≥ 1–49% of growth), or absent (no growth; 0%).

### Genome analysis

Gene clusters putatively involved in EPS biosynthesis were inferred through a BLAST search of the experimentally validated genes involved in the synthesis of known EPSs reported in Schmid et al. (2015), plus a hypothetical EPS biosynthetic cluster identified in *Pseudoalteromonas* sp. S3 (Yu et al. 2020), using the protein sequences. Genes were assigned to the best hits with the following thresholds: *e*-value < 1e − 20, sequence coverage > 50%, sequence similarity > 40%. The genomic context of the identified genes was manually checked to find other genes putatively involved in EPS biosynthesis.

A BLAST search, with the same thresholds, was performed to identify genes putatively involved in heavy metal resistance, using as queries the 378 experimentally confirmed protein sequences included in the BacMet database (http://bacmet.biomedicine.gu.se/) (Pal et al. 2014). Putative antibiotic resistance genes have been identified by the online Resistance Gene Identifier tool included in the CARD database (https://card.mcmaster.ca/) (Alcock et al. 2020) using the “Perfect, Strict and Loose hits” and “Low quality/coverage” options.

## Results

### Bacterial strain characterization

Gram-negative *Pseudoalteromonas* sp. S8-8 cells are asporogenic rods, strictly aerobic, and motile. Non-pigmented colonies on MA plates are circular (2–3 mm in diameter), convex, and shiny with entire edges. The strain grows at pH 5 to 9 (optimum pH 7–8) and at 4 to 30 °C (optimum 10–15 °C; no growth occurs at 37 °C). S8-8 can degrade chitin, starch, gelatin, Tween 80, and aesculin, whereas agar is not hydrolyzed. Acids are not produced from carbohydrates. Malate, mannitol, N-acetyl-glucosamine, maltose, gluconate, caprate, adipate, citrate, arabinose, and phenyl-acetate are not used by *Pseudoalteromonas* sp. S8-8 as the sole carbon and energy source. S8-8 does not reduce nitrate neither produce H_2_S, indole, and acetoin. No activity is recorded for several enzymes, such as catalase, β-galactosidase, urease, ornithine and lysine decarboxylase, arginine dihydrolase, and tryptophan deaminase. S8-8 is oxidase positive. Nalidixic acid, ampicillin, chloramphenicol, and polymixyn B inhibit the growth of S8-8. Conversely, it is resistant to penicillin G, tetracycline, tobramicin, and the vibriostatic agent O/129.

### Optimization of EPS production by Pseudoalteromonas sp. S8-8

**Fig. 1:**
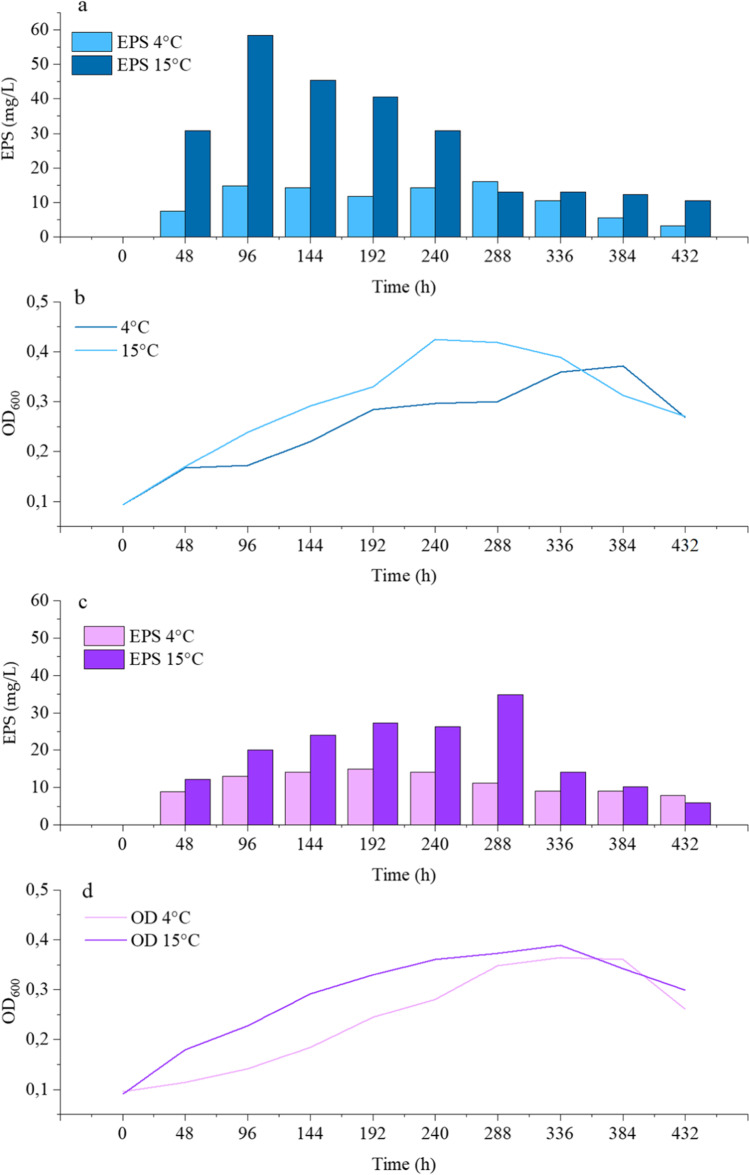
Effect of temperature on *Pseudoalteromonas* sp. S8-8 growth (lines) and EPS production (bars) during incubation at 4 °C and 15 °C in presence of glucose (**a**, **b**) and sucrose (**c**, **d**) (0.6%, w/v)

**Fig. 2:**
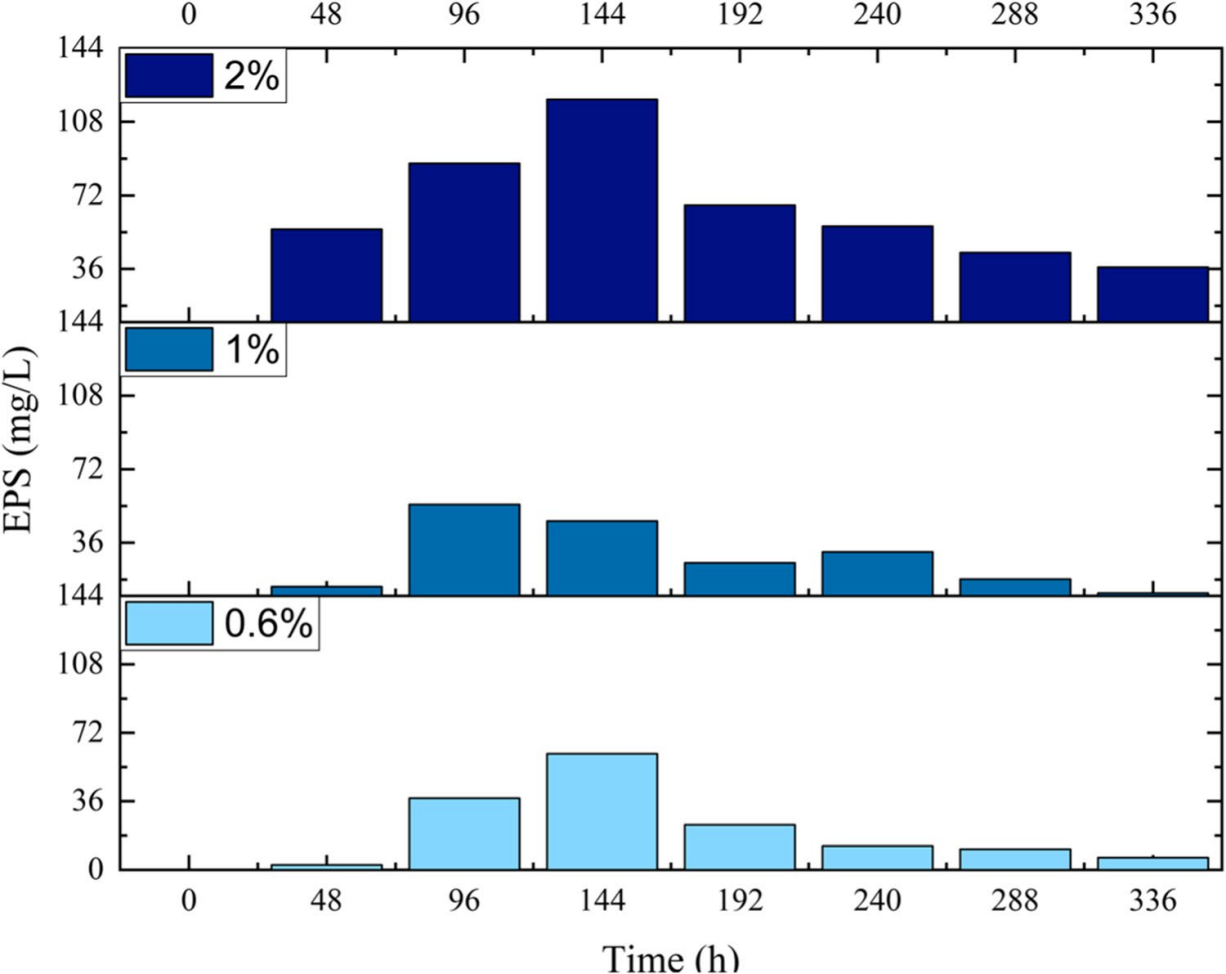
Influence of glucose concentration on EPS production by *Pseudoalteromonas* sp. S8-8 during growth at 15 °C, pH 7, and 3% of NaCl concentration

*Pseudoalteromonas* sp. S8-8 produced EPSs in the presence of glucose or sucrose. In both cases, higher EPS yields were obtained at 15 than 4 °C (Fig. 1a, c). At 15 °C, glucose allowed achieving the production of the highest amount (58.3 ± 11.0 mg/L) of exoproducts after 96 h (Fig. 1a), while the addition of sucrose in the culture medium determined the production of EPS amounts up to 34.8 ± 2.4 mg/L (after 288-h incubation) (Fig. 1c). EPS production generally increased in the exponential growth phase. Based on these results, glucose as substrate and the incubation temperature of 15 °C were retained for further analyses. An increase in EPS amounts was observed by shifting glucose concentration from 0.6 and 1% (60.9 mg/L after 96 h and 54.8 mg/L after 144 h, respectively) to 2% (118.8 mg/L after 144 h) when growing *Pseudoalteromonas* sp. S8-8 at 15 °C (Fig. 2).

**Fig. 3:**
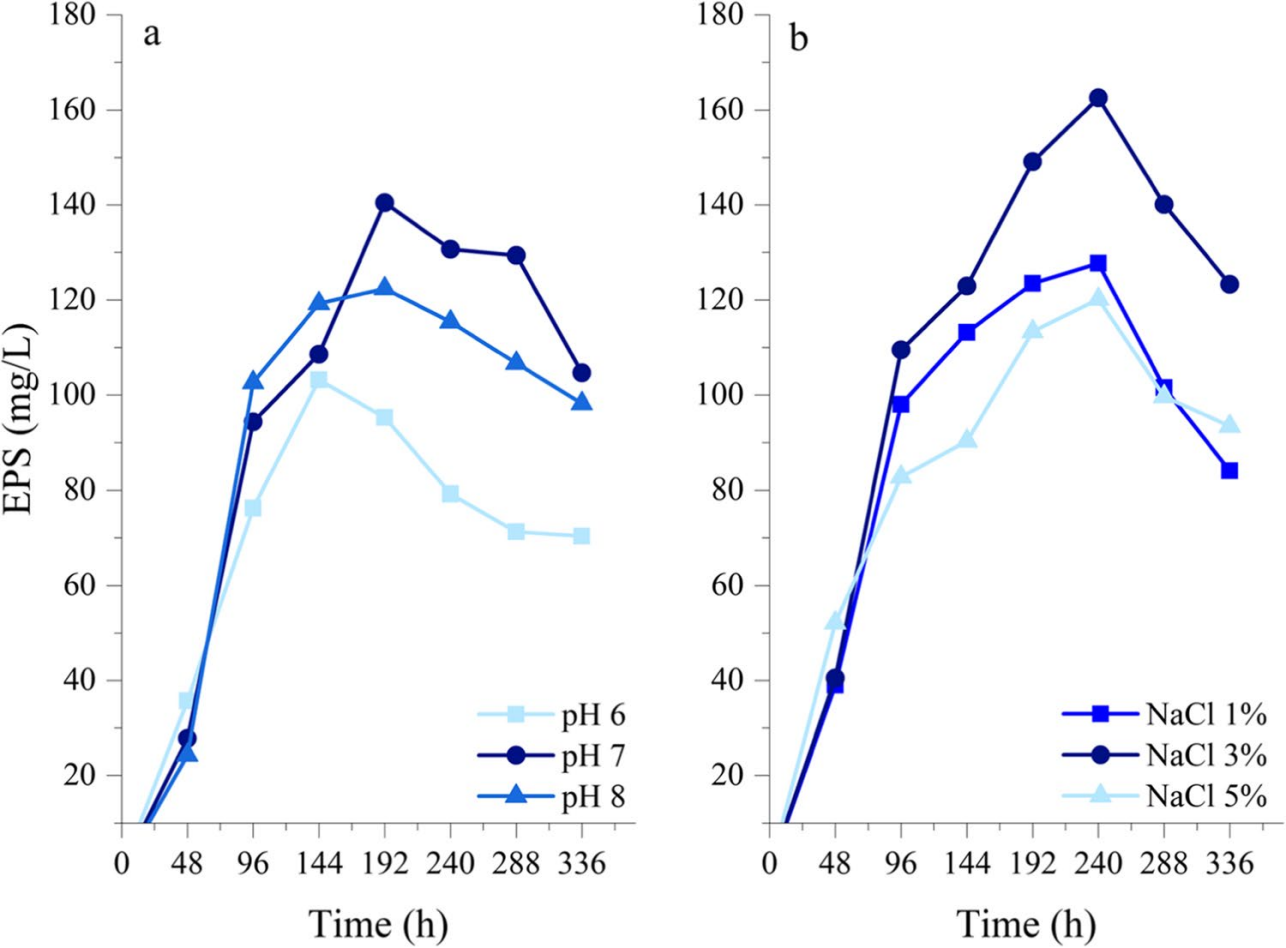
Effects of pH values and NaCl concentration on EPS production of *Pseudoalteromonas* sp. S8-8 during growth in the presence of glucose (2%, w/v) at 15 °C

**Fig. 4:**
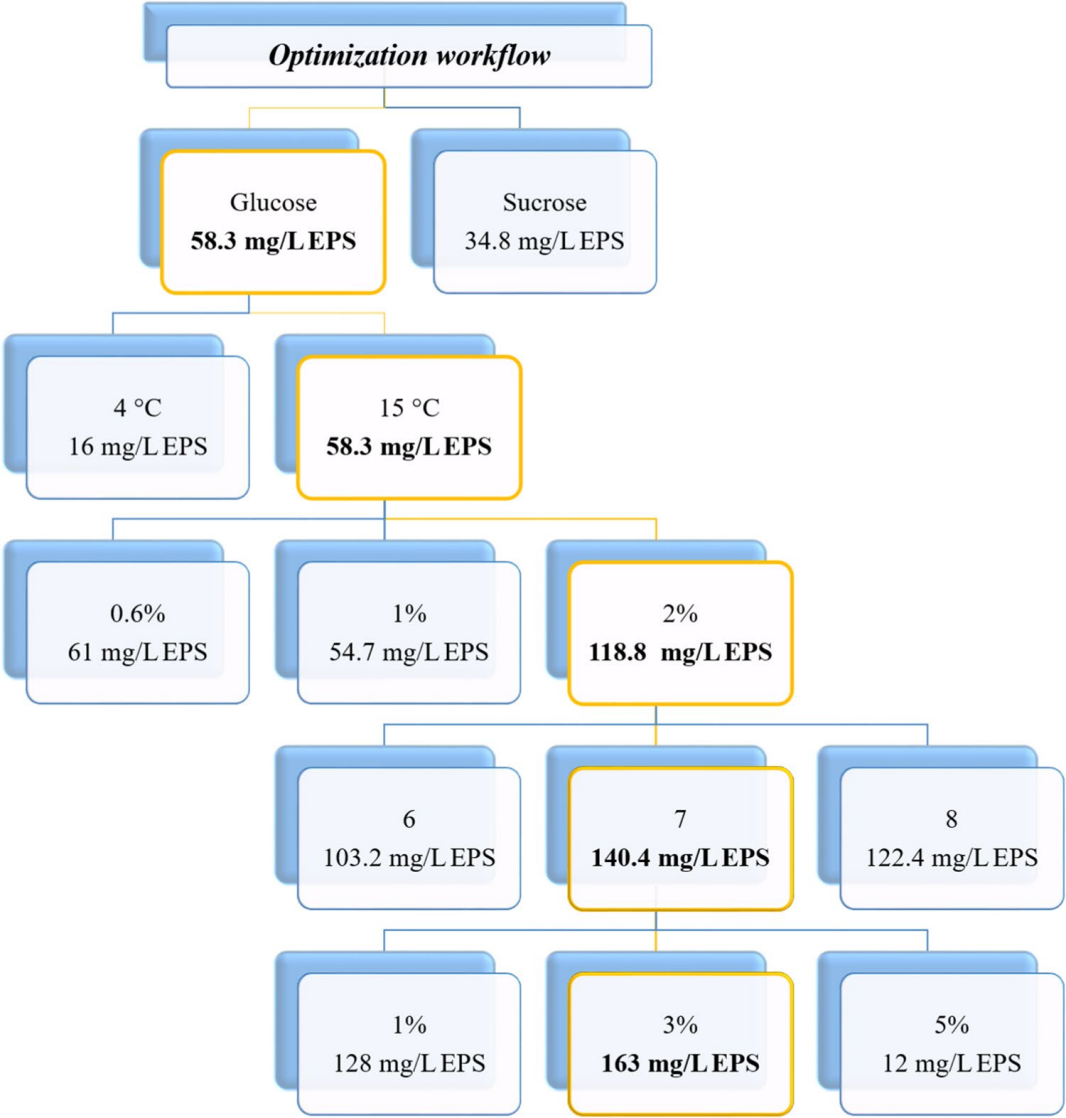
Optimization workflow for EPS production by *Pseudoalteromonas* sp. S8-8. Box in yellow represents the optimal conditions determined and maintained the step-by-step approach

To evaluate the effect of pH, *Pseudoalteromonas* sp. S8-8 was grown in the presence of glucose (final concentration 2%, w/v) at 15 °C. The pH value did not markedly influence the production of EPSs by S8-8. However, pH 7 was individuated as favorable for EPS production (up to 140.4 mg/L; Fig. 3a). Conversely, NaCl concentration severely affected both bacterial growth and EPS production (highest yield at 3% NaCl: 163 mg/L after 240 h) (Fig. 3b). The step-by-step approach described above led to individuate the optimal conditions for EPS production by *Pseudoalteromonas* sp. S8-8 (Fig. 4). Briefly, S8-8 produced up to 163 mg EPS/L in the exponential phase (after 120-h incubation) when growing at 15 °C and pH 7 in the presence of 2% (w/v) glucose and 3% (w/v) NaCl.

Glucose concentration strongly affected EPS production by *Pseudoalteromonas* sp. S8-8, as it was highlighted by the statistical analysis (*p* < 0.05). No significant differences were detected in EPS production during growth of the strain in the presence of the two carbon sources during growth at temperature of 4 °C, while differences of statistical concern occurred during growth at 15 °C, with EPS production that resulted significantly higher in the presence of glucose (*p* < 0.05). No significant differences were highlighted in EPS production by the strain during incubation at different conditions of pH and NaCl concentrations (*p* > 0.05).

### EPS extraction and chemical characterization

The optimal cultural conditions summarized in Fig. 4 were used to grow *Pseudoalteromonas* sp. S8-8 in batch culture. Prior to extract EPS, the phase of maximum EPS production was spectrophotometrically determined. Lyophilized exoproducts (characterized by compactness and scarce solubility in water) amounted for 89 mg/L. Carbohydrate, protein, and uronic acid contents in lyophilized and purified EPSs accounted for 35, 2.5 and 2.77%, respectively.

Main constituents of EPSs, as revealed by the HPAE-PAD analysis, were glucose:mannose:galactosamine:galactose (relative molar proportions of 1:0.7:0.5:0.4).

**Fig. 5:**
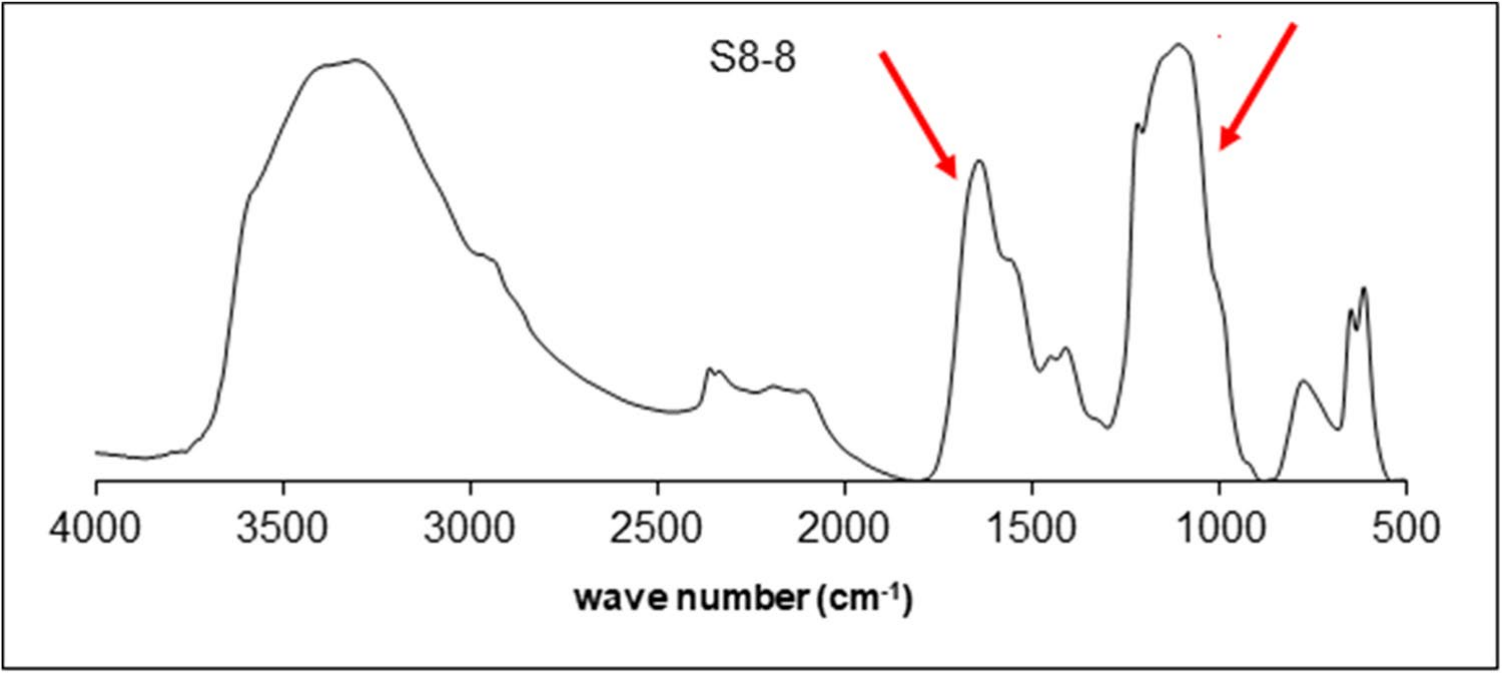
FT-IR spectra of EPSs produced by *Pseudoalteromonas* sp. S8-8

The FT-IR spectrum (Fig. 5) highlighted the occurrence of peaks visible between 1650 and 1050 cm^−1^, which are characteristic of an exopolysaccharide. The band at about 1050 cm^−1^, representing the bonds C-O and C–O–C, was typical of exopolysaccharides with an acidic nature. Amino sugars and proteins (1550 cm^−1^), and OH stretching (3300 cm^−1^) also occurred. The presence of methyl groups C-H was revealed by a minor band at 2900 cm^−1^. Sulfate content was about 20%. A peak at 1450 cm^−1^ (O–C = O bond) indicated the presence of uronic acids.

### Biotechnological potential of the EPSs

#### Emulsifying activity of EPSs

As shown in Table 1, the E_24_ percentages obtained from cultures of *Pseudoalteromonas* sp. S8-8 in the presence of hydrocarbons (except for tetradecane) were higher than those obtained by using synthetic surfactants as positive controls (i.e., Tween 80 and Triton X-100), with a maximum of 64%, 59%, and 68% in the presence of hexane, octane, and hexadecane, respectively.

**Table 1:** Emulsifying activity of EPS produced by *Pseudoalteromonas* sp. S8-8

		Emulsifying activity (%)			
Origine dell’EPS		Hexane	Octane	Hexadecane	Tetradecane
S8-8		64	59	68	22
Controls					
Tween 80		60	55	57	58
Triton X-100		60	58	58	60

#### EPSs as heavy metal chelating agents

*Pseudoalteromonas* sp. S8-8 showed a HM tolerance that was in the order Hg < Cd < Zn < Cu < Fe, and which was always higher in the medium amended with glucose. The strain completely (100% of growth) tolerated Fe up to 10,000 ppm, Cu up to 5000 ppm, Zn up to 2500 ppm, Cd up to 500 ppm, and Hg up to 100 ppm (Table 2).

**Table 2:**
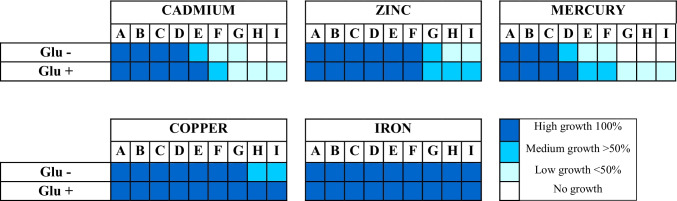
Heavy metal tolerance exhibited by *Pseudoalteromonas* sp. S8-8 in the presence and absence of glucose

A: 10 ppm; B: 50 ppm; C: 100 ppm; D: 500 ppm; E: 1000 ppm; F: 2500 ppm; G: 5000 ppm; H: 7500 ppm; I: 10000 ppm

#### EPSs as cryoprotective agents

**Fig. 6:**
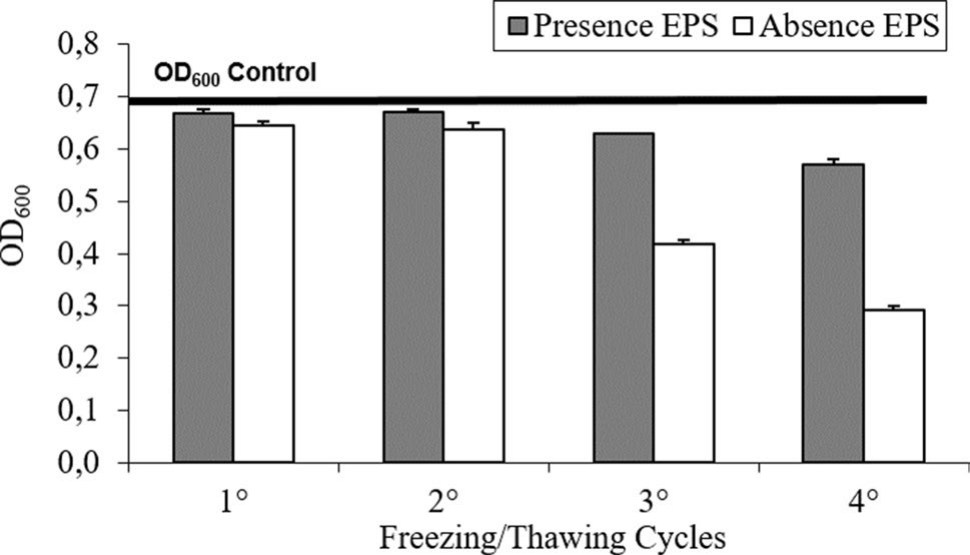
Growth of the EPS-producing *Pseudoalteromonas* sp. S8-8 after four freezing/thawing cycles. The black bar indicates OD values of MB inoculated with untreated bacteria (unfrozen)

A cryoprotective effect was strongly evidenced after the third and fourth freezing/thawing cycles in bacterial growth between EPS − and EPS + , reaching a 50% difference in cellular growth after the fourth cycle (Fig. 6).

### Genome analysis

#### EPS biosynthesis gene clusters in Pseudoalteromonas sp. S8-8 genome

The genome sequence of *Pseudoalteromonas* sp. S8-8 was analyzed to find gene clusters that might be putatively involved in EPS biosynthesis.

**Fig. 7:**
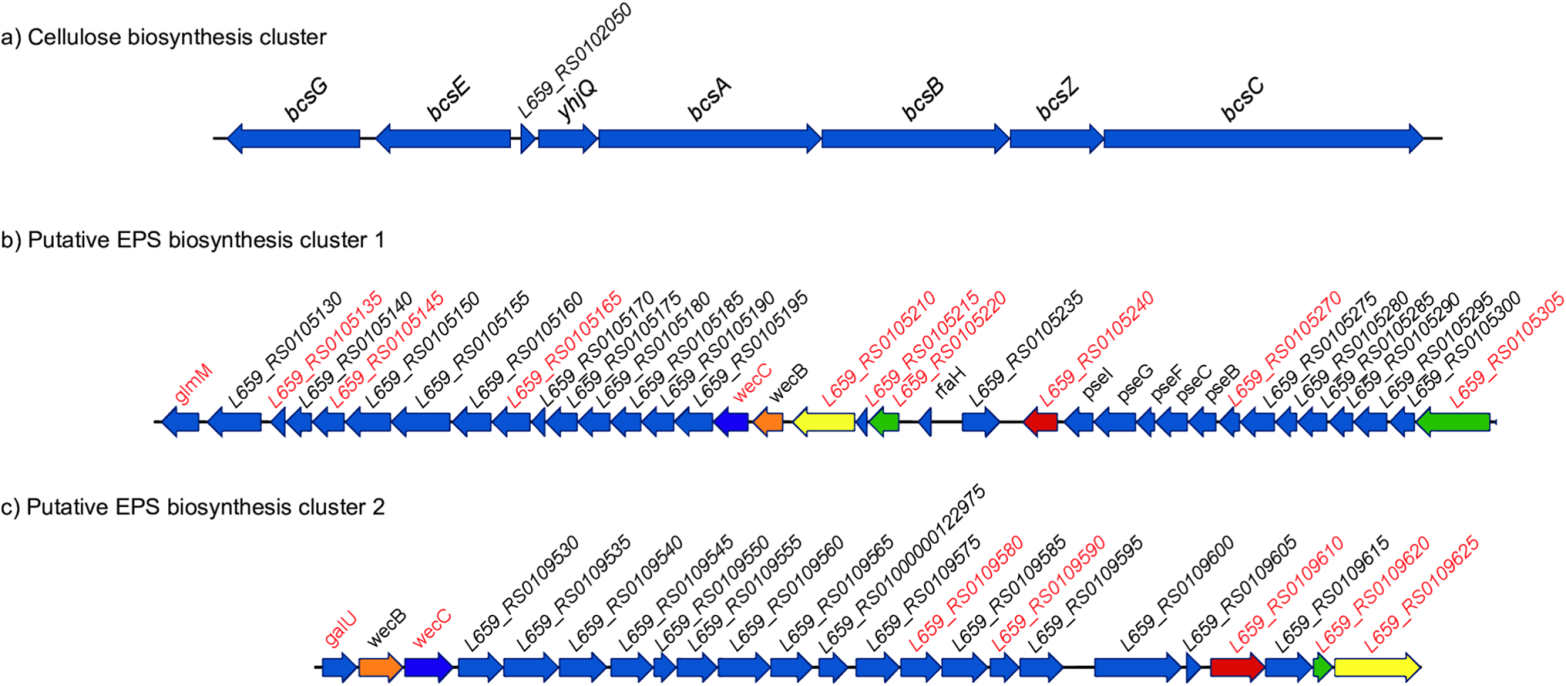
Genomic organization of the clusters putatively involved in EPS production identified in the *Pseudoalteromonas* sp. S8-8 genome. Genes with similarity with genes involved in EPS biosynthesis in other microorganisms (see Schmid et al. 2015) are highlighted in red. Paralogs genes are reported with the same color code

As shown in Fig. 7a and Table S1, a cluster for cellulose biosynthesis was identified. This cluster comprises seven open reading frames (ORF) very likely organized in two divergent operons and could be included in the *Escherichia coli*-like type of *bcs* operons, whose distinguishing features are the presence of a *yhjQ*, *bcsA*, *bcsB*, *bcsZ*, and *bcsC* operon, and of the *bcsE* and *bcsG* genes (and the absence of *bcsD)* (Römling and Galperin 2015) .

In addition, two regions of the genome contain several genes with similarity to genes involved in EPS biosynthesis in other non polar EPS-producing microorganisms (*Sphingomonas* sp., *Sinorhizobium meliloti*, *Escherichia coli*, *Pseudomonas aeruginosa*, *Xanthomonas campestris*, *Streptococcus pyrogenes*) (see Schmid et al. 2015) and with some of the genes included in a putative EPS biosynthetic cluster identified in a marine sediment isolate *Pseudoalteromonas* sp. S3 (Yu et al. 2020) (highlighted in red in Fig. 7b and c), and could be involved in the biosynthesis of the new identified EPSs (Fig. 7b and c, Table S1).

The first cluster (cluster 1), consisting of 36 ORFs, was located in contig 20, whereas the second one (cluster 2), including 23 ORFs, was identified in contig 33. The two clusters might be organized in transcriptional units (T.U.) (9 and 5 TU in cluster 1 and 2, respectively) on the basis of the length of the intergenic regions located between the different ORFs; in many cases, two consecutive ORFs overlaps at a different extent, suggesting a translation coupling, which, in turn, suggest that they belong to the same transcriptional unit.

The comparative analysis of the proteins encoded by the 59 ORFs revealed the existence of some paralogs (reported with the same color code in Fig. 7). In particular, the gene pair *wecB—wecC* was found in both clusters in the same relative order and sharing a degree of sequence similarity of about 80% spanning over the entire length of the amino acid sequence (see Table 3).

**Table 3:** Degree of sequence identity/similarity between the products of paralogous genes shared by contig 20 (cluster 1) and contig 33 (cluster 2) and putatively involved in the biosynthesis of EPS in *Pseudoalteromonas* sp. S8-8

		Cluster 1						Cluster 2				
		wecB	wecC	5210	5240	5305	5220	wecB	wecC	9265	9610	9620
Cluster 1	wecB							66.9				
	wecC								60.9			
	5210									21.0		
	5240										25.3	
	5305						13.9					21.4
	5220					34.7						18.6
Cluster 2	wecB	82.5										
	wecC		78.4									
	9265			48.9								
	9610				50.7							
	9620					50.8	40.8					

The two ORFs L659_RS0109610 (cluster 2) and L659_RS0105240 located on cluster 1 share a degree of sequence similarity of 50.7%, sufficiently high to suggest their origin by duplication of the same gene. Also, in this case, the similarity spans over the entire length of the amino acid sequences, even though some molecular rearrangements occurred in one or both genes.

Another pair of paralogs is represented by L659_RS0109625 (cluster 2) and L659_RS0105210 (cluster 1), sharing a degree of sequence similarity of 48.9% over the entire length of the proteins.

Lastly, L659_RS0109620 (in cluster 2) retrieved in a BLASTP search the products of two genes located in cluster 1 (i.e., L659_RS0105220 and L659_RS0105305). They share a degree of sequence similarity lower than that of the other paralogs; the analysis of the amino acid sequence of the three proteins revealed that they very likely are the outcome of the shuffling of different domains.

#### Heavy metal and antibiotic resistance genes in Pseudoalteromonas sp. S8-8 genome

A total of 69 genes coding for proteins putatively involved in heavy metal resistance have been identified in the *Pseudoalteromonas* sp. S8-8 genome (Table S2). They are mainly transporter proteins involved in resistance to several different heavy metals (including all the metals tested in this work) and transcriptional regulators, many of which are two component regulatory systems. Furthermore, two operons involved in nickel and mercury resistance have been identified (Table S2).

Regarding antibiotics, a total of 22 genes coding for putative resistance mechanisms were found (Table S3). Most of them are multidrug efflux pumps involved in resistance to different classes of antibiotics, but there are also three putative beta-lactamases, a dihydrofolate reductase putative involved in trimethoprim resistance, a glycopeptide resistance gene cluster, and a modified fluoroquinolone resistant *gyrB*.

## Discussion

In this work, different approaches were used trying to get much more information on the Antarctic strain *Pseudoalteromonas* sp. S8-8 and its ability to produce EPSs. Although several *Pseudoalteromonas* spp. strains are well-known EPS producers, the genus remains particularly interesting in the field, thanks to the great versatility and adaptability of its members. Previous studies have demonstrated intra-specific differences in the biosynthetic effectiveness, mainly depending on external parameters and strain origin (Caruso et al. 2018a,b; Corsaro et al. 2004; Mancuso Nichols et al. 2005a, b; Liu et al. 2012). Most of the *Pseudoalteromonas* strains so far investigated came from water samples, and minor reports are available for strains isolated from sediments (Kim and Yim 2007) . The origin of the producer and the different growth conditions are recognized influencing factors on the EPS yield and on the chemical structure of the molecules. Data obtained in this work confirm these aspects and partially support previous findings. Among carbon sources, *Pseudoalteromonas* sp. S8-8 showed preference for glucose rather than sucrose, as it grew and produced higher amounts of EPSs in the presence of it, thus differentiating from the previously reported *Pseudoalteromonas* sp. MER144 (Caruso et al. 2018b) and from other Antarctic strains previously investigated by the same procedure (Caruso et al. 2018a) which were more effective in the presence of sucrose. At the sugar final concentration of 2%, *Pseudoalteromonas* sp. S8-8 achieved a yield of 118.8 mg/L EPS, while *Pseudoalteromonas* sp. MER144 in the presence of sucrose at the same final concentration achieved a final EPS yield of 214.16 mg/L.

Independently from the used carbon source, it was observed that temperature was a determining factor on the production of EPS by S8-8 both in terms of quantity and production over time. While during incubation at 4 °C, the EPS yields were quite constant over time, during incubation at 15 °C the EPS production was higher, and in the presence of glucose as carbon source, it occurred anticipating the exponential phase. These aspects also contributed to differentiate strain S8-8 from other previously analyzed *Pseudoalteromonas* members (Mancuso Nichols et al., 2004), for which suboptimal temperatures (− 2 to 10 °C) were reported as an excellent condition for the production of EPS and the production occurred shortly before or in conjunction with the exponential phase (maximum yield of ca 100 mg EPS per gram dry weight of by the *Pseudoalteromonas* sp. CAM025) while during growth at 20 °C the production was ca 30-fold lower. Differently, the EPS production by other polar strains with different taxonomic affiliations is very variable. The Arctic strain *Polaribacter* SM1127 produced the highest EPS yield 2.11 g/L after growing at 15 °C with 30 g/L glucose (Sun et al. 2015) , while sponge-associated Antarctic bacteria produced EPS in a range from 143.7 to 396.7 mg/L during growth at 4 °C or 183.5 mg/L at 15 °C (Caruso et al., 2018a).

The best pH and NaCl concentration conditions retrieved for *Pseudoalteromonas* sp. S8-8 were in line with results formerly obtained by other Antarctic strains (Caruso et al. 2018a; Li et al. 2006) and *Pseudoalteromonas* spp. (Caruso et al. 2018b).

The chemical characterization carried out in this work suggested that the extract obtained is an exopolysaccharide containing amino sugars, as similarly reported for MER144. However, some structural differences exist between the extracts obtained from the two strains. Indeed, the carbohydrate content in the EPS of *Pseudoalteromonas* sp. S8-8 is higher (35%) than that found in *Pseudoalteromonas* sp. MER144 (18%), whereas the protein and uronic acid content is lower in *Pseudoalteromonas* sp. S8-8 than in *Pseudoalteromonas* sp. MER144. These parameters are in line with the EPS composition obtained from cold-adapted sponge-associated bacteria (Caruso et al. 2018a), whose EPSs chemical composition was as follows: carbohydrates, ranging from 15 to 28%; proteins, ranging from 2.08 to 8%; uronic acids, ranging from 3.2 to 11.9%. The same could be assumed in comparison with the Antarctic marine bacteria investigated by Mancuso Nichols et al. (2004, 2005a), whose EPSs showed a higher carbohydrate (74/50%) and lower protein (2/3%) amounts. However, the presence of uronic acids was evidenced by the FT-IR spectrum, as the 1000–1125 range is characteristic of uronic acid, O-acetyl ester linkage bond (Morillo Perez et al. 2008), and the sulfate content here retrieved is noticeably higher than all previously reported, as for example the 5% content reported for *Pseudoalteromonas* sp. CAM025 and CAM036 (Mancuso Nichols et al. 2004) or the 3.1% of *Pseudoalteromonas* sp. MER144 (Caruso et al. 2018b). The presence of amino sugars has been also highlighted by the FT-IR signal at 1550 cm^−1^ (Hamidi et al. 2019).

The presence of molecular groups conferring negative charge to the EPSs probably plays a role in the emulsifying and chelating functions of polymers, in agreement with previous data (Caruso et al. 2018a, b). Indeed, the EPS produced by *Pseudoalteromonas* sp. S8-8 showed a good emulsifying activity towards four hydrocarbons and enhanced the heavy metal tolerance of the strain in all the tested conditions, by tolerating higher concentration of the metal (e.g., cadmium and mercury) or by increasing the growth range at different metal concentrations (as it was the case of zinc, copper, iron).

A cryoprotective effect was detected also for the EPS produced by *Pseudoalteromonas* sp. S8-8, thus supporting its role in the survival of cold-adapted bacteria at subzero temperatures (Marx et al. 2009). Despite the EPS was produced at a higher temperature than that detected as optimal for *Pseudoalteromonas* sp. MER144, it exhibited good effectiveness at repeated freezing–thawing cycles. The strains showed a higher cryoprotective effect than those observed for *Winogradskyella* spp. strains and *Colwellia* sp. GW185 from Antarctic sponges (cryoprotective effect of 5, 11, and 25%; Caruso et al. 2018a), while an effect comparable to that highlighted for *Pseudoalteromonas* sp. MER 144 from Antarctic seawater (cryoprotective effect of 50%; Caruso et al. 2018b) and *Shewanella* sp. CAL606 from Antarctic sponges (cryoprotective effect of 50%; Caruso et al. 2018a) was observed.

The analysis of the *Pseudoalteromonas* sp. S8-8 genome revealed the existence, in addition to a complete cellulose biosynthetic cluster, of two long clusters of ORFs putatively involved in the biosynthesis of EPS. They include a total of 59 genes, the products of some of which shared a degree of similarity with other proteins involved in the EPS biosynthesis in other microorganisms sufficiently high to suggest their involvement in the *Pseudoalteromonas* sp. S8-8 EPS. The two clusters share some paralogous genes, which underwent more or less large genetic rearrangements after the duplication from the ancestor gene. In particular, a copy of the gene pair *wecB-wecC* organized in the same relative order is present on the two clusters; *wec*C has a similarity of 43% with *alg*D, which is involved in alginate biosynthesis in *Pseudomonas aeruginosa* (Schmid et al. 2015). They are surrounded by ORFs encoding glycosyltransferases and other proteins that might putatively be involved in the biosynthesis of EPS, but whose function is unknown. Hence, based on the available data, it is not possible to identify the metabolic pathway(s) leading to the synthesis of EPS in *Pseudoalteromonas* sp. S8-8, which might be very complex and would require several additional experiments, an issue which is beyond the scope of the present work.

In conclusion, in the search of new molecules of natural origin, the detection of new or under investigated producers needs to be accompanied by the understanding of its specific needs, the correct interpretation of the optimal conditions for the production of a bioproduct, which cannot stop at small-scale production systems but must be functional for subsequent development. The new genomic and bioinformatic methodologies come to the aid in this framework because they allow to identify everything that is not easily observable with a cultural approach if not following numerous experiments. A cultural approach to optimize net EPS yields, the subsequent elucidation of the chemical structure and functional properties of the product, and finally a genome mining approach made it possible to construct a detailed picture in the case of *Pseudoalteromonas* sp. S8-8. The strain has shown an excellent potential in biotechnological terms and has also confirmed how much the origin affects the specific needs and the consequent efficiency. The present work represents one of the few multidisciplinary approaches to the topic for bacteria from polar environments.

## Supplementary Information

Below is the link to the electronic supplementary material.

Supplementary file1 (PDF 571 KB)

## Data Availability

The data will be made available upon reasonable request.
