# Supplementary material for: Characterization of the exopolymer-producing Pseudoalteromonas sp. S8-8 from Antarctic sediment
Source: Appl Microbiol Biotechnol. 2022 Sep 26;106(21):7173–85. doi: 10.1007/s00253-022-12180-x (PMC9592659; doi:10.1007/s00253-022-12180-x)
Supplement: Supplementary file 1 — Supplementary file1 (PDF 571 KB) [file 253_2022_12180_MOESM1_ESM.pdf]

SUPPLEMENTARY MATERIALS

Journal *Applied Microbiology and Biotechnology*

Characterization of the exopolymer-producing *Pseudoalteromonas* sp. S8-8 from Antarctic sediment

Carmen Rizzo[a], Elena Perrin[b], Annarita Poli[c], Ilaria Finore[c], Renato Fani[b], Angelina Lo Giudice[d,e]

[a] *Stazione Zoologica "Anton Dohrn", Marine Biotechnology Department, Sicily Marine Centre, Villa Pace, Messina, Italy*

[b] *Department of Biology, University of Florence, Florence (Italy)*

[c] *Institute of Biomolecular Chemistry, National Research Council (ICB-CNR), Pozzuoli (NA), Italy*

[d] *Institute of Polar Sciences, National Research Council (CNR-ISP), Messina (Italy)*

[e] *Italian Collection of Antarctic Bacteria, National Antarctic Museum (CIBAN-MNA), Section of Messina, Messina (Italy)*

Corresponding author: Lo Giudice Angelina, Institute of Polar Sciences, National Research Council (CNR-ISP), Spianata San Raineri 86, 98122 Messina (Italy); Tel.: 0039 090 6015414; e-mail: angelina.logiudice@cnr.it

Legends

Supplementary Table 1 Putative EPS cluster identified in the *Pseudoalteromonas* sp. S8-8 genome

Supplementary Table 2 Genes putatively involved in heavy-metal resistance identified in the *Pseudoalteromonas* sp. S8-8 genome

Supplementary Table 3 Genes putatively involved in antibiotic resistance identified in the *Pseudoalteromonas* sp. S8-8 genome

Table S1. Putative EPS cluster identified in the *Pseudoalteromonas* sp. S8-8 genome

Cellulose biosynthesis cluster

Gene name	Protein_ID	Locus_tag	Description
bcsG	WP_024589430.1	L659_RS0102035	cellulose biosynthesis protein BcsG
bcsE	WP_024589431.1	L659_RS0102045	cellulose biosynthesis protein BcsE
	WP_007581346.1	L659_RS0102050	hypothetical protein
yhjQ	WP_024589432.1	L659_RS0102055	cellulose synthase operon protein YhjQ
bcsA	WP_024589433.1	L659_RS0102060	UDP-forming cellulose synthase catalytic subunit
bcsB	WP_024589434.1	L659_RS0102065	cellulose biosynthesis cyclic di-GMP-binding regulatory protein BcsB
bcsZ	WP_024589435.1	L659_RS0102070	cellulase
bcsC	WP_024589436.1	L659_RS0102075	BCSC C-terminal domain-containing protein

Putative EPS biosynthesis cluster

Gene name	Protein_ID	Locus_tag	Description	Similarity with	% Similarity
glmM	WP_024589807.1	L659_RS0105125	phosphoglucosamine mutase	ManB (*Escherichia coli*)	45%
	WP_024589808.1	L659_RS0105130	polysaccharide biosynthesis protein		
	WP_024589809.1	L659_RS0105135	sugar transferase	GumD (*Xantomonas campestris*)	54%
				WelB (*Sphingomonas* sp.)	52%
				ExoY (*Sinorhizobium meliloti*)	52%
	WP_024589810.1	L659_RS0105140	SDR family oxidoreductase		
	WP_024589811.1	L659_RS0105145	glycosyltransferase family 4 protein	WcaI (*Escherichia coli*)	42%
	WP_024589812.1	L659_RS0105150	alginate lyase family protein		
	WP_024589813.1	L659_RS0105155	bi-domain-containing oxidoreductase		
	WP_024589814.1	L659_RS0105160	phosphomannomutase		
	WP_196812464.1	L659_RS0105165	mannose-1-phosphate guanylyltransferase/mannose-6-phosphate isomerase	ManC (*Escherichia coli*)	76%
				AlgA (*Pseudomonas aeruginosa*)	72%
	WP_024589816.1	L659_RS0105170	acyltransferase		
	WP_024589817.1	L659_RS0105175	glycosyltransferase		
	WP_024589818.1	L659_RS0105180	glycosyltransferase		

	WP_024589819.1	L659_RS0105185	polysaccharide pyruvyl transferase family protein		
	WP_024589820.1	L659_RS0105190	hypothetical protein		
	WP_024589821.1	L659_RS0105195	oligosaccharide repeat unit polymerase		
wecC	WP_024589822.1	L659_RS0105200	UDP-N-acetyl-D-mannosamine dehydrogenase	AlgD (*Pseudomonas aeruginosa*)	43%
wecB	WP_024589823.1	L659_RS0105205	UDP-N-acetylglucosamine 2-epimerase (non-hydrolyzing)		
	WP_024589824.1	L659_RS0105210	polysaccharide biosynthesis tyrosine autokinase	GumC (*Xantomonas campestris*)	47%
				WelE (*Sphingomonas* sp.)	52%
				ExoP (*Sinorhizobium meliloti*)	41%
				Wzc (*Escherichia coli*)	62%
	WP_024589825.1	L659_RS0105215	low molecular weight phosphotyrosine protein phosphatase	Wzb (*Escherichia coli*)	62%
	WP_024589826.1	L659_RS0105220	polysaccharide biosynthesis/export family protein	WelD (*Sphingomonas* sp.)	55%
				ExoF (*Sinorhizobium meliloti*)	54%
				Wza (*Escherichia coli*)	67%
rfaH	WP_024589827.1	L659_RS0105230	transcription/translation regulatory transformer protein RfaH		
	WP_024589828.1	L659_RS0105235	MBL fold metallo-hydrolase		
	WP_081709142.1	L659_RS0105240	exopolysaccharide biosynthesis polyprenyl glycosylphosphotransferase	GumD (*Xantomonas campestris*)	57%
				WelB (*Sphingomonas* sp.)	60%
				ExoY (*Sinorhizobium meliloti*)	54%
pseI	WP_024589830.1	L659_RS0105245	pseudaminic acid synthase		
pseG	WP_024589831.1	L659_RS0105250	UDP-2,4-diacetamido-2,4,6-trideoxy-beta-L-altropyranose hydrolase		
pseF	WP_024589832.1	L659_RS0105255	pseudaminic acid cytidylyltransferase		
pseC	WP_024589833.1	L659_RS0105260	UDP-4-amino-4,6-dideoxy-N-acetyl-beta-L-altrosamine transaminase		
pseB	WP_024589834.1	L659_RS0105265	UDP-N-acetylglucosamine 4,6-dehydratase (inverting)		
	WP_024589835.1	L659_RS0105270	glycosyltransferase	ExoW (*Sinorhizobium meliloti*)	56%
				ExoO (*Sinorhizobium meliloti*)	55%
				WcaA (*Escherichia coli*)	63%
	WP_024589836.1	L659_RS0105275	FAD-dependent oxidoreductase		
	WP_024589837.1	L659_RS0105280	glycosyltransferase family 2 protein		
	WP_024589838.1	L659_RS0105285	EpsG family protein		
	WP_024589839.1	L659_RS0105290	hypothetical protein		

	WP_165721195.1	L659_RS0105295	oligosaccharide flippase family protein		
	WP_024589841.1	L659_RS0105300	hypothetical protein		
	WP_024589842.1	L659_RS0105305	SLBB domain-containing protein	GumB (*Xantomonas campestris*)	49%

Putative EPS biosynthesis cluster

Gene name	Protein_ID	Locus_tag	Description	Similarity with	% Similarity
galU	WP_024590377.1	L659_RS0109515	UTP--glucose-1-phosphate uridylyltransferase GalU	RmlA (*Sphingomonas* sp.)	46%
				ExoN (*Sinorhizobium meliloti*)	62%
wecB	WP_024590378.1	L659_RS0109520	UDP-N-acetylglucosamine 2-epimerase (non-hydrolyzing)		
wecC	WP_024590379.1	L659_RS0109525	UDP-N-acetyl-D-mannosamine dehydrogenase	AlgD (*Pseudomonas aeruginosa*)	44%
	WP_024590380.1	L659_RS0109530	VpsD family glycosyltransferase		
	WP_024590381.1	L659_RS0109535	lipopolysaccharide biosynthesis protein		
	WP_024590382.1	L659_RS0109540	VpsF family polysaccharide biosynthesis protein		
	WP_024590383.1	L659_RS0109545	hypothetical protein		
	WP_024590384.1	L659_RS0109550	GNAT family N-acetyltransferase		
	WP_024590385.1	L659_RS0109555	hypothetical protein		
	WP_024590386.1	L659_RS0109560	phenylacetate--CoA ligase family protein		
	WP_024590387.1	L659_RS0109565	glycosyltransferase family 4 protein		
	WP_155945819.1	L659_RS01000000122975	IS5 family transposase		
	WP_024590388.1	L659_RS0109575	hypothetical protein		
	WP_024590389.1	L659_RS0109580	glycosyltransferase family 4 protein	WcaL (*Escherichia coli*)	49%
	WP_024590390.1	L659_RS0109585	hypothetical protein		
	WP_024590391.1	L659_RS0109590	WecB/TagA/CpsF family glycosyltransferase	GumM (*Xantomonas campestris*)	47%
	WP_024590392.1	L659_RS0109595	hypothetical protein		
	WP_024590393.1	L659_RS0109600	choice-of-anchor A family protein		
	WP_024590394.1	L659_RS0109605	hypothetical protein		
	WP_024590395.1	L659_RS0109610	undecaprenyl-phosphate glucose phosphotransferase	WelB (*Sphingomonas* sp.)	49%
				ExoY (*Sinorhizobium meliloti*)	56%
				WcaJ (*Escherichia coli*)	60%
				GumD (*Xantomonas campestris*)	56%
	WP_024590396.1	L659_RS0109615	outer membrane beta-barrel protein		

WP_010554202.1	L659_RS0109620	polysaccharide export protein	ExoF (*Sinorhizobium meliloti*)	54%
WP_024590397.1	L659_RS0109625	polysaccharide biosynthesis tyrosine autokinase	ExoP (*Sinorhizobium meliloti*)	45%
			WelE (*Sphingomonas* sp.)	30%

Table S2. Genes putatively involved in heavy-metal resistance identified in the *Pseudoalteromonas* sp. S8-8 genome

**Gene_name in BacMet:**	**BacMet ID:**	**Code for:**	**Family:**	**Compound:**	**Description:**	**Reference:**	**Description in *Pseudolateromonas* S8_8:**	***Pseudolateromonas* S8_8 Protein ID:**	***Pseudolateromonas* S8_8 Locus Tag:**
mntH/yfeP	BAC0251	Membrane Transporter	NRAMP family	Manganese (Mn), Iron (Fe), Cadmium (Cd), Cobalt (Co), Zinc (Zn)	Divalent metal cation transporter MntH; H+-stimulated, divalent metal cation uptake system. Involved in manganese and iron uptake. Can also transport cadmium, cobalt, zinc and to a lesser extent nickel and copper. Involved in response to reactive oxygen.	Makui et al. 2000; Pubmed-10712688	Nramp family divalent metal transporter	WP_024589155.1	L659_RS0100030
ybtQ	BAC0433	Binding protein	ABC superfamily	Iron (Fe)	Permease and ATP-binding protein of yersiniabactin-iron ABC transport YbtQ	Fetherston et al. 1999; Pubmed-10231486	lipid A export permease/ATP-binding protein MsbA	WP_024589195.1	L659_RS0100300
fbpA	BAC0160	Binding protein	ATP-binding Cassette (ABC)-type ATPase superfamily. Bacterial solute-binding protein 1 family	Iron (Fe), Gallium (Ga)	Fe(3+)-binding/Iron-utilization periplasmic protein FbpA; Part of the ABC transporter complex FbpABC involved in Fe3+ ions import. This protein specifically binds Fe3+ and is involved in its transmembrane transport	Anderson et al. 2004; Pubmed-15342592	extracellular solute-binding protein	WP_024589224.1	L659_RS0100550

wtpC	BAC0604	Enzyme	ABC transporter superfamily. ATPase family	Tungsten (W), Molybdenum (Mo)	Molybdate/tungstate import ATP-binding protein WtpC (ATPase). Part of the ABC transporter complex WtpABC involved in molybdate/tungstate import. Responsible for energy coupling to the transport system.	Bevers et al. 2006; Pubmed-16952940	ABC transporter ATP-binding protein	WP_024589368.1	L659_RS0101525
corB	BAC0643	Enzyme	Cation transport ATPase (P-type) family	Cobalt (Co), Magnesium (Mg)	Magnesium-transporting ATPase, P-type 1; Mediates magnesium influx to the cytosol.	Gibson et al. 1991; Pubmed-1779764, Hmiel et al. 1989; Pubmed-2548998	CNNM domain-containing protein	WP_007581396.1	L659_RS0101855
acn	BAC0003	Enzyme	Aconitase family	Iron (Fe)	Aconitate hydratase, Acn	Wong et al. 1999; Pubmed-9864233	aconitate hydratase AcnA	WP_024589528.1	L659_RS0102815
recG	BAC0356	Enzyme	Contains 1 DEAD/DEAH box helicase domain	Chromium (Cr), Tellurium (Te), Selenium (Se)	ATP-dependent DNA helicase RecG. It is involved in repairing DNA damage caused by chromate or its derivatives. Can confer resistant to tellurite, selenite but not arsenite, paraquat or hydrogen peroxide.	Miranda et al. 2005; Pubmed-16105671, Decorosi et al. 2009; Pubmed-19768364	ATP-dependent DNA helicase RecG	WP_024589556.1	L659_RS0103005
perO	BAC0612	Enzyme	ArsB/NhaD permeases family	Molybdenum (Mo), Tungsten (W), Vanadium (V)	PerO is involved in molybdate uptake. In addition to importing molybdate, PerO probably importssulfate, tungstate, and vanadate.	Gisin et al. 2010; Pubmed-20851900	SLC13 family permease	WP_024589585.1	L659_RS0103240
copY/tcrY	BAC0084	Regulator	BlaI transcriptional regulatory family	Copper (Cu)	CopA and CopB upstream repressor CopY, regulates the cop operon; Binds to DNA in complex with Zn2+, repressing the transcription of the CopYZAB operon. Exchange of the bound zinc by two copper ions delivered by CopZ Cu1+-bound form causes release of CopY from the promoter, leading to the transcription of the operon	Strusak et al. 1997; Pubmed-9083014, Wunderli-Ye et al. 1999; Pubmed-10362527	BlaI/MecI/CopY family transcriptional regulator	WP_007580531.1	L659_RS0103655

copB	BAC0626	Enzyme	Cation transport ATPase (P-type) family. Type IB subfamily	Copper (Cu)	Copper resistance protein B. It mediates copper resistance by its sequestration in the outer membrane. Required for the copper-inducible expression of copper resistance; part of the copper resistance determinant system.	Mellano et al. 1988; Pubmed-3372485, Cha et al. 1991; Pubmed-1924351	copper resistance protein B	WP_024589685.1	L659_RS0104020
pcoB	BAC0304	Enzyme	CopB family	Copper (Cu)	opper resistance protein PcoB; required for the copper-inducible expression of copper resistance; located in cell outer membrane; it's peripheral membrane protein	Brown et al. 1995; Pubmed-8594334	copper resistance protein B	WP_024589685.1	L659_RS0104020
copA	BAC0077	Enzyme	Multicopper oxidase (mco) family	Copper (Cu), Silver (Ag)	Encode for copper uptake ATPases; also resistant to Ag+ ; periplasmic protein; Probably involved in copper and silver Export. Mediates copper resistance by sequestration of copper in the periplasm along with the copper-binding protein CopC. May have oxidase activity.	Cha and Cooksey 1991; Pubmed-1924351, Outten et al. 2001; Pubmed-11399769	copper resistance system multicopper oxidase	WP_024589686.1	L659_RS0104025
mmco	BAC0480	Enzyme	Multicopper oxidase (mco) family	Copper (Cu)	Mmco is required for copper resistance in Mycobacterium tuberculosis. It acts by oxidation of toxic Cu(I) in the periplasm.	Rowland et al. 2013; Pubmed-23772064	copper resistance system multicopper oxidase	WP_024589686.1	L659_RS0104025
pcoA	BAC0303	Enzyme	Multi-copper oxidase family (CopA subfamily)	Copper (Cu)	Copper resistance protein PcoA; required for the copper-inducible expression of copper resistance. May have oxidase activity; part of the copper resistance determinant system (pcoABCDRSE)	Brown et al. 1995; Pubmed-8594334	copper resistance system multicopper oxidase	WP_024589686.1	L659_RS0104025

czcB	BAC0120	Efflux	Membrane fusion protein (MFP) family	Cadmium (Cd), Zinc (Zn), Cobalt (Co)	Cobalt-zinc-cadmium resistance protein CzcB; Membrane fusion protein bridges inner and outer cell membranes (in periplasm); CzcA and CzcB together would act in zinc efflux nearly as effectively as the complete czc efflux system (CzcABC). The CzcB protein is thought to funnel zinc cations to the CzcA transport protein.	Nies et al. 1989; Pubmed-2678100, Waidner et al. 2002; Pubmed-12426358	efflux RND transporter periplasmic adaptor subunit	WP_024589741.1	L659_RS0104535
czrB	BAC0131	Efflux	RND superfamily, MFP family	Zinc (Zn), Cadmium (Cd)	czrB involved in cadmium and zinc resistance; part of the czrSRCBA resistance operon	Hassan et al. 1999; Pubmed-10570969, Kuroda et al. 1999; Pubmed-10229265	efflux RND transporter periplasmic adaptor subunit	WP_024589741.1	L659_RS0104535
cnrA	BAC0203	Efflux	RND superfamily, AcrB/AcrD/AcrF family	Cobalt (Co), Nickel (Ni)	Nickel and cobalt resistance protein CnrA. The products of the genes cnrA, cnrB, and cnrC are likely to form a membrane-bound protein complex catalyzing an energy-dependent efflux of Ni2+ and Co2+. The mechanism of action of the CnrCBA complex may be that of a proton/cation antiporter.	Liesegang et al. 1993; Pubmed-8380802, Grass et al. 2000; Pubmed-10671463	CusA/CzcA family heavy metal efflux RND transporter	WP_024589742.1	L659_RS0104540
czcA	BAC0119	Efflux	RND superfamily, AcrB/AcrD/AcrF family	Cadmium (Cd), Zinc (Zn), Cobalt (Co)	Cobalt-zinc-cadmium resistance protein; the basic inner membrane transport protein; Has a low cation transport activity for cobalt, it is essential for the expression of cobalt, zinc, and cadmium resistance. CzcA and CzcB together would act in zinc efflux nearly as effectively as the complete CZC efflux system (CzcABC).	Nies et al. 1989; Pubmed-2678100	CusA/CzcA family heavy metal efflux RND transporter	WP_024589742.1	L659_RS0104540
czrA	BAC0130	Regulator	RND superfamily, AcrB/AcrD/AcrF family	Zinc (Zn), Cadmium (Cd)	A repressor for thr czr operon; part of the czrSRCBA resistance operon	Valencia et al. 2013; Pubmed-23578014; Hassan et al. 1999; Pubmed-10570969, Kuroda et al. 1999; Pubmed-10229265	CusA/CzcA family heavy metal efflux RND transporter	WP_024589742.1	L659_RS0104540

nccA	BAC0549	Efflux	RND superfamily, AcrB/AcrD/AcrF family	Nickel (Ni), Cobalt (Co), Cadmium (Cd)	Nickel-cobalt-cadmium resistance protein NccA. Component of the NCC cation-efflux system (nccYXHCBAN) that confers resistance to nickel, cobalt and cadmium. May form a membrane tunnel, which allows ion transport across the membrane.	Schmidt et al. 1994; Pubmed-7961470	CusA/CzcA family heavy metal efflux RND transporter	WP_024589742.1	L659_RS0104540
nczA	BAC0267	Regulator	RND superfamily, AcrB/AcrD/AcrF family	Nickel (Ni), Cobalt (Co), Zinc (Zn)	Nickel-cobalt-zinc resistance protein nczA; part of the nczCBA operon; The nczCBA operon is induced maximally by Ni2+ and Co2+, moderately by Zn2+ but not by Cd2+. NczCBA transports Ni2+ Co2+ and probably Zn2+	Valencia et al. 2013; Pubmed-23578014	CusA/CzcA family heavy metal efflux RND transporter	WP_024589742.1	L659_RS0104540
actP	BAC0570	Membrane transporter/Enzyme	Sodium:solute symporter (SSF) family	Tellurium (Te)	Acetate Permease (ActP) is responsible for tellurite uptake and resistance in cells of the facultative phototroph Rhodobacter capsulatus.	Borghese et al. 2010; Pubmed-19966028	cation acetate symporter, partial	WP_024589746.1	L659_RS0104585
zur/yjbK	BAC0470	Regulator	Fur family	Zinc (Zn)	Zinc uptake regulation protein zur; Acts as a negative controlling element, employing Zn2+ as a cofactor to bind the operator of the repressed genes (znuACB). Zinc uptake regulation protein.	Patzer et al. 1998; Pubmed-9680209, Patzer et al. 2000; Pubmed-10816566, Li et al. 2009; Pubmed-19552825	transcriptional repressor	WP_007580267.1	L659_RS0104860
corD	BAC0644	Unknown	Contains 1 apaG domain	Cobalt (Co), Magnesium (Mg)	mutations in apaG/corD give a phenotype of low-level Co2+ resistance. They also decrease Mg2+ efflux but not influx via the CorA Mg2+ transport system.	Gibson et al. 1991; Pubmed-1779764, Hmiel et al. 1989; Pubmed-2548998	Co2+/Mg2+ efflux protein ApaG	WP_002960839.1	L659_RS0105085

ruvB	BAC0293	Enzyme	Malic enzymes family	Chromium (Cr), Cetylpyridinium Chloride (CPC) [class: Quaternary Ammonium Compounds (QACs)], Dodine [class: Acetate], 2-nitroimidazole [class: imidazole]	It is a fragment of ruvB gene product. It is a oxidoreductase from malic enzyme family.	Decorosi et al. 2009; Pubmed-19768364	malate dehydrogenase	WP_007582028.1	L659_RS0105690
dsbB	BAC0137	Enzyme	DsbB family	Cadmium (Cd), Mercury (Hg)	Disulfide oxidoreductase; The dsbB gene codes for a transmembrane protein that is responsible for reoxidizing the periplasmic dsbA-encoded disulfide oxidoreductase; Required for disulfide bond formation in some periplasmic proteins such as PhoA or OmpA	Stafford et al. 1999; Pubmed-10234837	disulfide bond formation protein DsbB	WP_007584117.1	L659_RS0106005
aioR/aoxR	BAC0022	Regulator	Contains 1 sigma-54 factor interaction and 1 sigma-5factor interaction domain	Arsenic (As)	Transcriptional regulator. Part of a two-component As(III) responsive system (AioSR)	Kashyap et al. 2006; Pubmed-16428412; Drewniak et al. 2013 Pubmed-23454063, Muller et al. 2003; Pubmed-12486049	sigma 54-interacting transcriptional regulator	WP_024590064.1	L659_RS0107075
corR	BAC0089	Regulator	Contains 1 sigma-54 factor interaction domain	Copper (Cu)	Sigma-54 dependent DNA-binding response regulator CorR; corSR copper-responsive two component system that induces carotenoid production. It also regulates three multicopper oxidases: cuoA, cuoB, cuoC, and two P-type ATPases: copA and copB.	Sanchez-Sutil et al. 2013; Pubmed-23874560	sigma 54-interacting transcriptional regulator	WP_024590064.1	L659_RS0107075

zraR/hydH	BAC0467	Regulator	Contains 1 response regulatory domain, 1 sigma-54 factor interaction domain	Zinc (Zn)	ranscriptional regulatory protein ZraR; Member of the two-component regulatory system ZraS/ZraR. When activated by ZraS it acts in conjunction with sigma-54 to regulate the Expression of zraP. Positively autoregulates the Expression of the zraSR operon.	Leonshartsberger et al. 2001; Pubmed-11243806	sigma 54-interacting transcriptional regulator	WP_024590064.1	L659_RS0107075
ruvB	BAC0355	Enzyme	RuvB family	Chromium (Cr), Tellurium (Te), Selenium (Se)	ATP-dependent DNA helicase RuvB. It is involved in repairing DNA damage caused by chromate or its derivatives. Can confer resistant to tellurite, selenite but not arsenite, paraquat or hydrogen peroxide.	Miranda et al. 2005; Pubmed-16105671, Decorosi et al. 2009; Pubmed-19768364	Holliday junction branch migration DNA helicase RuvB	WP_007580382.1	L659_RS0110325
cusB	BAC0108	Efflux	Membrane fusion protein(MFP) family	Copper (Cu), Silver (Ag)	Part of a cation efflux system (CusA, CusB, CusC and CusF) that mediates resistance to copper and silver; Belongs to the membrane fusion protein (MFP) family	Franke et al. 2003; Pubmed-12813074	efflux RND transporter periplasmic adaptor subunit	WP_024590587.1	L659_RS0111150
silB	BAC0342	Efflux	Membrane fusion protein (MFP) family	Silver (Ag)	ilB, a membrane fusion protein that brings together the inner and outer membranes of Gram-negative bacteria; Component of the sil cation-efflux system (silABC) that confers resistance to silver	Gupta et al. 1999; Pubmed-9930866	efflux RND transporter periplasmic adaptor subunit	WP_024590587.1	L659_RS0111150
cusA/ybdE	BAC0107	Efflux	RND superfamily; AcrB/AcrD/AcrF family	Copper (Cu), Silver (Ag)	Part of a cation efflux system (CusA, CusB, CusC and CusF) that mediates resistance to copper and silver; located in cell inner membrane; Belongs to the AcrB/AcrD/AcrF family	Franke et al. 2003; Pubmed-12813074	efflux RND transporter permease subunit	WP_024590588.1	L659_RS0111155

silA	BAC0341	Efflux	AcrB/AcrD/AcrF family	Silver (Ag)	SilA is a inner membrane chemiosmotic, cation/proton antiporter; Component of the sil cation-efflux system (silABC) that confers resistance to silver	Gupta et al. 1999; Pubmed-9930866	efflux RND transporter permease subunit	WP_024590588.1	L659_RS0111155
pstB	BAC0316	Enzyme	ABC transporter superfamily. Phospahte importer family	Arsenic (As)	Phosphate transporting ATPase; Part of the ABC transporter complex PstSACB involved in phosphate import. Responsible for energy coupling to the transport system.	Willsky et al. 1980; Pubmed-6998959, Willsky et al. 1980; Pubmed-6998957, Surin et al. 1985; Pubmed-3881386	phosphate ABC transporter ATP-binding protein PstB	WP_007586464.1	L659_RS0111180
tupC	BAC0607	Enzyme	ABC superfamily, ATPase family	Tungsten (W)	ATPase component of tungstate ABC transporter	Makdessi et al. 2001; Pubmed-11292832	phosphate ABC transporter ATP-binding protein PstB	WP_007586464.1	L659_RS0111180
fieF/yiip	BAC0167	Efflux	Cation diffusion facilitator (CDF) transporter family	Iron (Fe), Zinc (Zn), Cobalt (Co), Cadmium (Cd), Nickel (Ni)	Iron-efflux transporter responsible for iron detoxification. Also able to transport Zn2+ in a proton-dependent manner. The CDF protein, FieF, is mainly a ferrous iron detoxifying protein but also mediated some resistance against other divalent metal cations such as Zn(II), Co(II), Cd(II), and Ni(II) in W. metallidurans or Escherichia coli.	Grass et al. 2005; Pubmed-15549269, Munkelt et al. 2004; Pubmed-15547276	cation diffusion facilitator family transporter	WP_081709160.1	L659_RS0111460
fbpB	BAC0161	Binding protein	ATP-binding Cassette (ABC)-type ATPase superfamily. Binding-protein-dependent transport system permease family. FbpB subfamily	Iron (Fe), Gallium (Ga)	Fe(3+)-transport system permease protein FbpB; Part of the ABC transporter complex FbpABC involved in Fe3+ ions import. This protein specifically binds Fe3+ and is involved in its transmembrane transport	Anderson et al. 2004; Pubmed-15342592	iron ABC transporter permease	WP_024590633.1	L659_RS0111575

fbpC	BAC0162	Binding protein	ATP-binding Cassette (ABC)-type ATPase superfamily	Iron (Fe), Gallium (Ga)	Fe(3+) ions import ATP-binding protein FbpC; Part of the ABC transporter complex FbpABC involved in Fe3+ ions import. Responsible for energy coupling to the transport system	Anderson et al. 2004; Pubmed-15342592	ABC transporter ATP-binding protein	WP_024590634.1	L659_RS0111580
modC	BAC0600	Enzyme	ABC superfamily, Molybdate importer family	ungsten (W), Molybdenum (Mo)	olybdenum ABC transporter, ATPase subunit of the ModABC complex. Part of the ABC transporter complex ModABC involved in molybdenum import. Responsible for energy coupling to the transport system.	Kazarov et al. 2013; Pubmed-23913324	ABC transporter ATP-binding protein	WP_024590634.1	L659_RS0111580
troA	BAC0399	Binding protein	ABC superfamily	Zinc (Zn), Manganese (Mn), Iron (Fe)	Periplasmic zinc-binding protein TroA; encodes the solute binding protein (SBP), Part of an ATP-driven transport system TroABCD for zinc. Substrate-binding protein involved in the transport of zinc across the cytoplasmic membrane.	Hazlett et al. 2003; Pubmed-12668673	NUDIX domain-containing protein	WP_010553288.1	L659_RS0111960
fecE	BAC0164	Membrane Transporter	ABC transporter superfamily	Nickel (Ni), Cobalt (Co)	Fe(3+) dicitrate transport ATP-binding protein FecE; Part of the binding-protein-dependent transport system for citrate-dependent Fe3+. Probably responsible for energy coupling to the transport system	Stoof et al. 2010; Pubmed-20643857	ABC transporter ATP-binding protein	WP_024590706.1	L659_RS0112080
fecD	BAC0163	Enzyme	Binding-protein-dependent transport system permease family. FecCD subfamily	Nickel (Ni), Cobalt (Co)	Fe(3+) dicitrate transport system permease protein FecD; Part of the binding-protein-dependent transport system for citrate-dependent Fe3+. Probably responsible for the translocation of the substrate across the membrane.	Stoof et al. 2010; Pubmed-20643857	iron ABC transporter permease	WP_024590707.1	L659_RS0112085

dsbC	BAC0138	Enzyme	Thioredoxin family. DsbC subfamily	Copper (Cu)	Disulfide isomerase; DsbC rearranges incorrect disulfide bonds formed by dsbA during oxidative protein folding under non-stress conditions. DsbC, a periplasmic thiol-disulfide oxidoreductase, appears to function as a disulfide isomerase both in vitro and in vivo. In vitro, DsbC has been shown to rearrange non-native disulfides in well studied isomerization substrates such as BPTI and RNase A. In vivo, DsbC is required for full activity of a handful of proteins containing at least one non-consecutive disulfide bond.	Hiniker et al. 2005; Pubmed-16087673	bifunctional protein-disulfide isomerase/oxidoreductase DsbC	WP_007583768.1	L659_RS0112175
nrsR	BAC0288	Regulator	Contains 1 response regulatory domain	Nickel (Ni)	OmpR subfamily protein NrsR/RppA/Rre33	Lopez-Maury et al. 2002; Pubmed-11849552	response regulator transcription factor	WP_007375447.1	L659_RS0113680
modB	BAC0599	Enzyme	ABC superfamily, binding-protein-dependent transport system permease family. CysTW subfamily	Tungsten (W), Molybdenum (Mo)	Molybdenum ABC transporter, permease protein. Part of the ModABC transport system. Responsible for the translocation of the substrate across the membrane. modB gene encodes the integral membrane protein ModB, which builds the membrane channel of the ModABC transporter.	Kazarov et al. 2013; Pubmed-23913324	molybdate ABC transporter permease subunit	WP_002959821.1	L659_RS0113715
nikE	BAC0274	Binding protein	ABC transporter superfamily. Nickel importer family	Nickel (Ni)	Nickel import ATP-binding protein NikE; Part of the ABC transporter complex NikABCDE involved in nickel import. Responsible for energy coupling to the transport system.	Navarro et al. 1993; Pubmed-7934931	ABC transporter ATP-binding protein	WP_024590982.1	L659_RS0114210

nikD	BAC0273	Binding protein	ABC transporter superfamily. Nickel importer family	Nickel (Ni)	Nickel import ATP-binding protein NikD; Part of the ABC transporter complex NikABCDE involved in nickel import. Responsible for energy coupling to the transport system. Part of the Nik operon (NikABCDE)	Navarro et al. 1993; Pubmed-7934931	ATP-binding cassette domain-containing protein	WP_007580952.1	L659_RS0114215
nikC	BAC0272	Membrane Transporter	Binding-protein-dependent transport system permease family. OppBC subfamily	Nickel (Ni)	Nickel transport system permease protein NikC; Transmembrane protein; Involved in a nickel transport system, probably translocates nickel through the bacterial inner membrane. Part of the Nik operon (NikABCDE)	Navarro et al. 1993; Pubmed-7934931	ABC transporter permease subunit	WP_024590983.1	L659_RS0114220
nikB	BAC0271	Membrane Transporter	Binding-protein-dependent transport system permease family. OppBC subfamily	Nickel (Ni)	Nickel transport system permease protein NikB; Transmembrane protein; Involved in a nickel transport system, probably translocates nickel through the bacterial inner membrane. Part of the Nik operon (NikABCDE)	Wu et al. 1991; Pubmed-1743519, Navarro et al. 1993; Pubmed-7934931	ABC transporter permease	WP_007580956.1	L659_RS0114225
nikA	BAC0270	Binding protein	Bacterial solute-binding protein 5 family	Nickel (Ni)	Nickel-binding periplasmic protein nikA; Involved in a nickel transport system, represents the nickel binder. Part of the Nik operon (NikABCDE), Solute-binding protein	Wu et al. 1991; Pubmed-1743519, De Pina et al. 1995; Pubmed-7867647	ABC transporter substrate-binding protein	WP_010553755.1	L659_RS0114230

ALU1-P	BAC0489	Enzyme	QueC family protein	Aluminium (Al)	Fragment of the ALU1 resistance gene called ALU1-P but the frament has the potential to confer tolerance to aluminium . It has 65% identity in amino acid level with the protein of YbaX gene in Escherichia coli, and 51.1% identity with YB91 Haein hypothetical protein of HI1191 gene in Haemophilus influenzae.	Jo et al. 1997; Pubmed-9367855	7-cyano-7-deazaguanine synthase QueC	WP_007583902.1	L659_RS011453
G2alt	BAC0490	Enzyme	PP-loop ATPase superfamily/QueC family	Aluminium (Al)	The ATPase activity of G2alt requires Mg2+ and Na+ ions, while Zn2+ and Al3+ stimulate the activity, Cd2+ and Ag+ reduces the activity and Li+, Cu2+ and Co2+ inhibits the activity.	Beris et al. 2011; Pubmed-21887649	7-cyano-7-deazaguanine synthase QueC	WP_007583902.1	L659_RS0114530
pbrT	BAC0302	Membrane Transporter	Contains 1 cytochrome C domain	Lead (Pb)	A lead (Pb++) uptake protein PbrT	Borremans et al. 2001; Pubmed-11544228	cytochrome c/FTR1 family iron permease	WP_024591062.1	L659_RS0114915
corC	BAC0088	Efflux	UPF0053 family. Contains 2 CBS domains.	Cobalt (Co), Magnesium (Mg)	Magnesium and cobalt efflux protein CorC; Plays a role in the transport of magnesium and cobalt ions	Gibson et al. 1991; Pubmed-1779764, Hmiel et al. 1989; Pubmed-2548998	CNNM family magnesium/cobalt transport protein CorC	WP_007585597.1	L659_RS0115420
cutE/lnt	BAC0115	Enzyme	CN hydrolase (Apolipoprotein N-acyltransferase subfamily)	Copper (Cu)	Apolipoprotein N-acyltransferas, it attaches a fatty acid to apo-nlpE (cutF)	Rogers et al. 1991; Pubmed-1938881	apolipoprotein N-acyltransferase	WP_024591113.1	L659_RS0115425
merR	BAC0680	Regulator	Contains 1 HTH merR-type DNA-binding domain	Mercury (Hg)	Mediates the mercuric-dependent induction of mercury resistance operon. In the absence of mercury MerR represses transcription by binding tightly to the mer operator region; when mercury is present the dimeric complex binds a single ion and becomes a potent transcriptional activator, while remaining bound to the mer site.	Laddaga et al. 1987; Pubmed-3037534	Hg(II)-responsive transcriptional regulator	WP_024591202.1	L659_RS0116085
merT	BAC0690	Membrane Transporter	MerT family	Mercury (Hg)	Mercuric ion transport protein.	Kalyaeva et al. 2001; Pubmed-11642118	mercury transporter	WP_024591203.1	L659_RS0116095

merP	BAC0674	Membrane Transporter	Contains 1 HMA domain	Mercury (Hg)	Periplasmic mercuric ion binding protein.	Kalyaeva et al. 2001; Pubmed-11642118	mercury resistance system periplasmic binding protein MerP	WP_024591204.1	L659_RS0116100
merC	BAC0664	Membrane Transporter	MerC family	Mercury (Hg)	Mercuric resistance protein MerC. This protein is located in the inner membrane and mediates mercury transport into the cytoplasm.	Reniero et al. 1995; Pubmed-8529897, Reniero et al. 1998; Pubmed-9479042	organomercurial transporter MerC	WP_024591205.1	L659_RS0116105
merA	BAC0224	Enzyme	Class-I pyridine nucleotide-disulfide oxidoreductase family	Mercury (Hg), Phenylmercury Acetate [class: Organo-mercury]	Mercury reductase enzyme, merA; resistance to Hg2+ in bacteria appears to be governed by a specialized system which includes mercuric reductase. MerA protein is responsible for volatilizing mercury as Hg0.	Gupta et al. 1999; Pubmed-10559175	mercury(II) reductase	WP_024591206.1	L659_RS0116110
ybtP	BAC0432	Binding protein	ABC superfamily	Iron (Fe)	Fe3+-Yersiniabactin uptake transporter, YbtP; inner membrane lipoprotein	Fetherston et al. 1999; Pubmed-10231486	ATP-binding cassette domain-containing protein	WP_024591375.1	L659_RS0117510
chrA	BAC0063	Efflux	Chromate ion transporter (CHR) family	Chromium (Cr)	Chromate (CrO4) transport protein ChrA; Responsible for the inducibility of the resistance	Cervantes et al. 1990; Pubmed-2152903	chromate efflux transporter	WP_024591376.1	L659_RS0117520
srpC	BAC0152	Efflux	Chromate Ion Transporter (CHR) family	Chromium (Cr)	A homologue of chrA. SrpC is not a sulfate transporter. Functions as a transporter that extrudes chromate ions from cytoplasm under sulfur-deficient conditions.	Aguilar-Barajas et al. 2012; Pubmed-22805806	chromate efflux transporter	WP_024591376.1	L659_RS0117520
pmrC	BAC0485	Membrane protein	Contains 1 sulfatase, 1 DUF1705 domain	Iron (Fe)	Putative cytoplasmic membrane protein pmrC. PmrA-activated pmrC gene is required for Fe(III) resistance.	Nishino et al. 2006; Pubmed-16803591, Hyytiainen et al. 2003; Pubmed-14617142	phosphoethanolamine--lipid A transferase	WP_024591427.1	L659_RS0117830

dsbA	BAC0136	Enzyme	Thioredoxin family. DsbA subfamily.	Cadmium (Cd), Zinc (Zn), Mercury (Hg)	Disulfide oxidoreductase; Required for disulfide bond formation in some periplasmic proteins such as PhoA or OmpA. Acts by transferring its disulfide bond to other proteins and is reduced in the process. DsbA is reoxidized by DsbB. It is required for pilus biogenesis	Rensing et al. 1997; Pubmed-9098080, Bardwell et al. 1991; Pubmed-1934062; Stafford et al. 1999; Pubmed-10234837	thiol:disulfide interchange protein DsbA/DsbL	WP_010555409.1	L659_RS0118000
fptA	BAC0168	Porin	General Bacterial Porin family (GBP) superfamily. TonB-dependent receptor family	Iron (Fe), Cobalt (Co), Nickel (Ni), Gallium (Ga)	Fe(3+)-pyochelin receptor FptA; High-affinity outer membrane receptor required for the transport of Fe3+-pyochelin	Braud et al. 2009; Pubmed-19329644	TonB-dependent siderophore receptor	WP_024591487.1	L659_RS0118425
fpvA	BAC0169	Porin	General Bacterial Porin family (GBP) superfamily. TonB-dependent receptor family	Manganese (Mn), Iron (Fe), Cobalt (Co), Zinc (Zn), Nickel (Ni), Copper (Cu), Cadmium (Cd), Gallium (Ga)	Ferripyoverdine receptor A (fpvA); Receptor for the siderophore ferripyoverdine; Located in cell outer membrane	Hannauer et al. 2012; Pubmed-22187978, Braud et al. 2009; Pubmed-19207567	TonB-dependent siderophore receptor	WP_024591487.1	L659_RS0118425
crdR	BAC0095	Regulator	Contains 1 Response_reg domain, 1 Trans_reg_C domain	Copper (Cu)	Response regulator; part of two-component regulatory system crdR/crdS	Waidner et al. 2005; Pubmed-15968080	response regulator transcription factor	WP_007376647.1	L659_RS0118715

pmrA	BAC0487	Regulator	Contains response regulatory domain	Iron (Fe)	Member of the PmrA-PmrB two-component system required in regulation of virulence in bacteria. It is involved in controlling of bacterial response to external pH and iron and is crucial for bacterial virulence. PmrA-PmrB system is also required for resistance to the peptide antibiotic polymyxin B and other cationic antimicrobial peptides. It is activated in vivo by direct or indirect means and regulates genes that modify lipopolysaccharide, aiding survival in host (and non-host) environments. Unphosphorylated PmrA represses extracellular enzyme genes. Phosphorylation of PmrA by PmrB relieves such repression, which leads to activation of extracellular enzyme genes. Phosphorylated PmrA seems to repress expression of the pmrCAB operon.	Hyytiainen et al. 2003; Pubmed-14617142; Wosten et al. 2000; Pubmed-11051552	response regulator transcription factor	WP_007376647.1	L659_RS0118715

pmrB	BAC0488	Regulator	Contains a sensor kinase domain	Iron (Fe)	Member of the PmrA-PmrB two-component system required in regulation of virulence in bacteria. Functions as a sensor protein kinase which is autophosphorylated at a histidine residue and transfers its phosphate group to PmrA in response to external pH and iron and is crucial for bacterial virulence. PmrA-PmrB system is also required for resistance to the peptide antibiotic polymyxin B and other cationic antimicrobial peptides. It is activated in vivo by direct or indirect means and regulates genes that modify lipopolysaccharide, aiding survival in host (and non-host) environments.	Hyytiainen et al. 2003; Pubmed-14617142, Wosten et al. 2000; Pubmed-1105155	two-component sensor histidine kinase	WP_024591520.1	L659_RS0118720
arsR	BAC0588	Regulator	Contains 1 HTH arsR-type DNA-binding domain	Arsenic (As)	Arsenite inducible repressor ArsR	Neyt et al. 1997; Pubmed-9006011	metalloregulator ArsR/SmtB family transcription factor	WP_024591540.1	L659_RS0118905
yfeB	BAC0440	Membrane Transporter	ABC transporter superfamily	Iron (Fe), Manganese (Mn)	Chelated iron transport system membrane protein YfeB; Part of an ATP-driven transport system YfeABCD for chelated iron	Bearden et al. 1998; Pubmed-9495751	heme ABC transporter ATP-binding protein	WP_029769942.1	L659_RS0119135
troB	BAC0400	Binding protein	ABC superfamily	Zinc (Zn), Manganese (Mn), Iron (Fe)	Zinc transport system ATP-binding protein TroB; cytoplasmic membrane permeases; Part of an ATP-driven transport system TroABCD for zinc	Hazlett et al. 2003; Pubmed-12668673	heme ABC transporter ATP-binding protein	WP_029769942.1	L659_RS0119140
actP	BAC0012	Enzyme	Cation transport ATPase (P-type) family	Copper (Cu), Sodium acetate [class: Acetate]	Copper-transporting P-type ATPase; Involved in copper efflux; Transcriptionally regulated by HmrR in response to Cu+ ions.	Reeve et al. 2002; Pubmed-11936079	heavy metal translocating P-type ATPase	WP_024591617.1	L659_RS0119575

cadA/yvgW	BAC0055	Enzyme	Cation transport ATPase (P-type) family	Cadmium (Cd), Zinc (Zn)	Cadmium efflux ATPase, has two domains (phosphatase, aspartyl kinase); Cadmium, zinc and cobalt-transporting ATPase; Couples the hydrolysis of ATP with the transport of cadmium, zinc and cobalt out of the cell	Herrman et al. 1999; Pubmed-10417643, Nucifera et al. 1989; Pubmed-2524829, Lee et al. 2001; Pubmed-11282588	heavy metal translocating P-type ATPase	WP_024591617.1	L659_RS0119575
copA	BAC0078	Enzyme	Cation transport ATPase (P-type) family	Copper (Cu)	Involved in copper export; The copZA operon is activated by CueR and indirectly repressed by YfmP	Banci et al. 2003; Pubmed-14514665, Outten et al. 2001; Pubmed-11399769	heavy metal translocating P-type ATPase	WP_024591617.1	L659_RS0119575
corT/coaT	BAC0092	Enzyme	Cation transport ATPase (P-type) family	Cobalt (Co)	Cation-transporting ATPase E1-E2 ATPase, CorT	Rutherford et al. 1999; Pubmed-10464323, Garcia-Dominquez et al. 2000; Pubmed-10692354	heavy metal translocating P-type ATPase	WP_024591617.1	L659_RS0119575
ctpC	BAC0098	Enzyme	Cation transport ATPase (P-type)family. Type IB subfamily	Manganese (Mn), Zinc (Zn)	Probable manganese/zinc-exporting P-type ATPase	Padilla-Benavides et al. 2013; Pubmed-23482562	heavy metal translocating P-type ATPase	WP_024591617.1	L659_RS0119575
ctpD	BAC0099	Enzyme	Cation transport ATPase (P-type)family. Type IB subfamily	Cobalt (Co), Nickel (Ni)	Probable cobalt/nickel-exporting P-type ATPase; Involved in heavy metal homeostasis. Probably exports nickel and cobalt ions out of the cell.	Raimunda et al. 2012; Pubmed-22591178	heavy metal translocating P-type ATPase	WP_024591617.1	L659_RS0119575
ctpG	BAC0100	Enzyme	Cation transport ATPase (P-type)	Copper (Cu)	Cation-transporting ATPase G; Response to copper ion	Liu et al. 2007; Pubmed-17143269, Sakamoto et al. 2010; Pubmed-20395270	heavy metal translocating P-type ATPase	WP_024591617.1	L659_RS0119575
ctpV	BAC0101	Enzyme	Cation transport ATPase (P-type)	Copper (Cu)	Copper-exporting P-type ATPase V; Necessary for copper homeostasis and likely functions as a copper exporter. Also required for full virulence.	Ward et al. 2010; Pubmed-20624225	heavy metal translocating P-type ATPase	WP_024591617.1	L659_RS0119575
cueA	BAC0102	Enzyme	Cation transport ATPase (P-type)	Copper (Cu), Silver (Ag)	Copper transporter CueA; Periplasmic protein	Adaikkalam et al. 2002; Pubmed-12213931	heavy metal translocating P-type ATPase	WP_024591617.1	L659_RS0119575

czcP	BAC0124	Enzyme	Cation transport ATPase (P-type) family	Cadmium (Cd), Zinc (Zn), Cobalt (Co)	P(IB4)-type ATPases; CzcP seemed to enhance metal resistance but relies on the action of the P(IB2)-type ATPases such as zntA, cadA, pbrA etc.	Scherer et al. 2009; Pubmed-19602147	heavy metal translocating P-type ATPase	WP_024591617.1	L659_RS0119575
golT	BAC0183	Enzyme	Cation transport ATPase (P-type) family	Copper (Cu), Gold (Au)	Cation transporting P-type ATPase; belongs to the gold-responsive, gol regulon; In the absence of CopA, GolS becomes responsive to copper, inducing the expression of golT, which functionally substitutes CopA for cytosolic copper export	Espariz et al. 2007, Pubmed-17768242	heavy metal translocating P-type ATPase	WP_024591617.1	L659_RS0119575
hpcopA	BAC0617	Enzyme	Cation transport ATPase (P-type) family, Type IB subfamily	Copper (Cu)	Involved in copper export.	Ge et al. 1995; Pubmed-7752900, Ge et al. 1996; Pubmed-8961555	heavy metal translocating P-type ATPase	WP_024591617.1	L659_RS0119575
nia	BAC0269	Enzyme	Cation transport ATPase (P-type) family	Iron (Fe), Nickel (Ni)	P1B-5-ATPase; Has role in detoxification of Fe (II)+ and Ni (II)+ under free living conditions as well as in symbiosis.	Zielazinski et al. 2013; Pubmed-24056637	heavy metal translocating P-type ATPase	WP_024591617.1	L659_RS0119575
pbrA	BAC0298	Enzyme	Cation transport ATPase (P-type) family	Lead (Pb)	pbrA, a P-type ATPase (involved in efflux); involved in Pb(II) resistance; part of the lead resistance determinant pbr operon (pbrABCD) which combines functions involved in uptake, efflux, and accumulation of Pb(II)	Borremans et al. 2001; Pubmed-11544228	heavy metal translocating P-type ATPase	WP_024591617.1	L659_RS0119575
silP	BAC0346	Enzyme	Cation transport ATPase (P-type) family (Type IB subfamily)	Silver (Ag)	Silver exporting P-type ATPase silP; Component of the sil cation-efflux system that confers resistance to silver; silP can participate in resistance to Ag+ individually; located in cell membrane.	Gupta et al. 1999; Pubmed-9930866	heavy metal translocating P-type ATPase	WP_024591617.1	L659_RS0119575

tcrB	BAC0383	Enzyme	Cation transport ATPase (P-type) family	Copper (Cu)	The transferable, plasmid-localized Copper resistance efflux ATPase, TcrB	Hasman te al. 2002; Pubmed-11959576, Hasman et al. 2005; Pubmed-16151212	heavy metal translocating P-type ATPase	WP_024591617.1	L659_RS0119575
ziaA	BAC0455	Enzyme	Cation transport ATPase (P-type) family, Type IB subfamily	Zinc (Zn)	Zinc-transporting ATPase ziaA	Thelwell et al. 1998: Pubmed-9724772	heavy metal translocating P-type ATPase	WP_024591617.1	L659_RS0119575
zntA/yhhO	BAC0461	Enzyme	Cation transport ATPase (P-type) family	Lead (Pb), Cadmium (Cd), Zinc (Zn)	Lead, cadmium, zinc and mercury-transporting ATPase ZntA; Involved in export of lead, cadmium, zinc and mercury; located in inner cell membrane.	ensing et al. 1997; Pubmed-9405611, Beard et al. 1997; Pubmed-9364914	heavy metal translocating P-type ATPase	WP_024591617.1	L659_RS0119575
copR	BAC0083	Regulator	Contains 1 response regulatory domain	Copper (Cu)	Transcriptional activator protein CopR; Member of the two-component regulatory system CopS/CopR. Involved in the activation of copper resistance gene operon CopABCD. Phosphorylation of CopR by CopS would convert it into an active state to induce Expression of the cop operon by binding to a specific site on the cop operon promoter (cop box) and possibly by facilitating the binding of RNA polymerase to the cop promoter. CopR also binds to the chromosomally encoded cop operon promoter. May also be involved in basic copper metabolism.	Mills et al. 1993; Pubmed-8449873	heavy metal response regulator transcription factor	WP_024591667.1	L659_RS0120015

cusR/ylcA	BAC0111	Regulator	Contains 1 response regulatory domain	Copper (Cu), Silver (Ag)	Member of the two-component regulatory system CusS/CusR. Activates the Expression of cusCFBA and plasmid pRJ1004 gene pcoE in response to increasing levels of copper ions, and probably silver ions. Can also increase the basal-level Expression of copper resistance gene operon pcoABCD; located in cytoplasm	Munson et al. 2000; Pubmed-11004187	heavy metal response regulator transcription factor	WP_024591667.1	L659_RS0120015
pcoR	BAC0308	Regulator	Contains 1 response regulatory domain	Copper (Cu)	Transcriptional regulatory protein PcoR; member of a two-component regulatory system pcoS/pcoR. Involved in the activation of copper resistance gene operon pcoABCD by binding to a specific site on the cop operon promoter (copper box). part of the copper resistance determinant system (pcoABCDRSE)	Rouch & Brown. 1997; Pubmed-9141682	heavy metal response regulator transcription factor	WP_024591667.1	L659_RS0120015
silR	BAC0347	Regulator	Contains 1 response regulatory domain.	Silver (Ag)	silR encodes an transcriptional regulator; Component of the sil cation-efflux system that confers resistance to silver. Probable member of a two-component regulatory system SilS/SilR.	Gupta et al. 1999; Pubmed-9930866	heavy metal response regulator transcription factor	WP_024591667.1	L659_RS0120015
copS	BAC0637	Regulator	Contains histidine kinase domain	Copper (Cu)	Member of the two-component regulatory system CopS/CopR. May also be involved in basic copper metabolism.	Hu et al. 2009; Pubmed-19557345	heavy metal sensor histidine kinase	WP_024591668.1	L659_RS0120020

silS	BAC0348	Regulator	Contains 1 HAMP domain and 1 histidine kinase domain.	Silver (Ag)	silS encodes a membrane sensor kinase; Component of the sil cation-efflux system that confers resistance to silver. Probable member of a two-component regulatory system SilS/SilR. May activate SilR by phosphorylation	Gupta et al. 1999; Pubmed-9930866	heavy metal sensor histidine kinase	WP_024591668.1	L659_RS0120020
chrR	BAC0538	Enzyme	NADH_dh2 family	Chromium (Cr), Iron (Fe), Hydrogen Peroxide (H2O2) [class: Peroxides], 2,6-dichloroindophenol [class: Phenolic Compounds]	A soluble quinone reductase of Pseudomonas putida that defends against H2O2. ChrR is a dimeric flavin mononucleotide-binding flavoprotein that is able to catalyze a full reduction of Cr(VI) to Cr(III). ChrR may also contribute to the survival of P. putida in the environment by preventing redox cycling of soluble quinones. Confers resistance to Pottasium Ferricyanide (Fe++ salt) as well.	Gonzalez et al. 2005; Pubmed-15840577, Ackerley et al. 2004; Pubmed-14766567, Park et al. 2000; Pubmed-10788340	NAD(P)H-dependent oxidoreductase	WP_024591699.1	L659_RS0120300
cusS	BAC0112	Regulator	Contains 1 HAMP domain and 1 histidine kinase domain	Copper (Cu), Silver (Ag)	Sensor kinase CusS; Member of the two-component regulatory system CusS/CusR. Copper ion sensor. Could also be a silver ion sensor. Activates CusR by phosphorylation; located in inner cell membrane	Munson et al. 2000; Pubmed-11004187	heavy metal sensor histidine kinase	WP_024591713.1	L659_RS0120415
czcS	BAC0126	Regulator	Contains 1 HAMP domain, 1 histidine kinase domain	Cadmium (Cd), Zinc (Zn), Cobalt (Co)	Sensor protein CzcS; Member of the two-component regulatory system CzcS/CzcR involved in the control of cobalt, zinc and cadmium homeostasis. Probably activates CzcR by phosphorylation.	Van der Lelie et al. 1997; Pubmed-9044283	heavy metal sensor histidine kinase	WP_024591713.1	L659_RS0120415

irlS	BAC0198	Regulator	Contains 1 histidine kinase domain, 1 HAMP domain	Cadmium (Cd), Zinc (Zn)	Sensor protein IrlS; Member of the two-component regulatory system IrlR/IrlS. May be involved in invasion of eukaryotic cells and heavy-metal resistance. Probably activates IrlR by phosphorylation.	Jones et al. 1997; Pubmed-9393784	heavy metal sensor histidine kinase	WP_024591713.1	L659_RS0120415
pcoS	BAC0309	Regulator	Contains 1 HAMP domain, 1 histidine kinase domain	Copper (Cu)	Sensor protein PcoS; member of a two-component regulatory system PcoS/PcoR. Activate PcoR by phosphorylation; part of the copper resistance determinant system (pcoABCDRSE)	Brown et al. 1995; Pubmed-8594334	heavy metal sensor histidine kinase	WP_024591713.1	L659_RS0120415
czcR	BAC0125	Regulator	Contains 1 response regulatory domain	Cadmium (Cd), Zinc (Zn), Cobalt (Co)	Transcriptional activator protein CzcR; Member of the two-component regulatory system CzcS/CzcR involved in the control of cobalt, zinc and cadmium homeostasis	Nies et al. 1992; Pubmed-1459958	heavy metal response regulator transcription factor	WP_024591714.1	L659_RS0120420
irlR	BAC0197	Regulator	Contains 1 response regulatory domain	Cadmium (Cd), Zinc (Zn)	Transcriptional activator protein IrlR; Member of the two-component regulatory system IrlR/IrlS. May be involved in invasion of eukaryotic cells and heavy-metal resistance	Jones et al. 1997; Pubmed-9393784	heavy metal response regulator transcription factor	WP_024591714.1	L659_RS0120420
dnaK	BAC0133	Chaperone	Heat shock protein 70 family	Copper (Cu)	Chaperone protein DnaK	Graubner et al. 2007; Pubmed-17215254	molecular chaperone DnaK	WP_024591803.1	L659_RS0121050
cusC/ylcB	BAC0109	Efflux	RND Superfamily, OMP family	Copper (Cu), Silver (Ag)	Part of a cation efflux system (CusA, CusB, CusC and CusF) that mediates resistance to copper and silver	Franke et al. 2003; Pubmed-12813074	efflux transporter outer membrane subunit	WP_024591829.1	L659_RS0121220
silC	BAC0343	Efflux	Outer Membrane Protein (OMP) family	Silver (Ag)	SilC is an outer membrane lipoprotein; Component of the sil cation-efflux system (silABC) that confers resistance to silver	Gupta et al. 1999; Pubmed-9930866	efflux transporter outer membrane subunit	WP_024591829.1	L659_RS0121220

srpC	BAC0374	Efflux	RND Superfamily, OMP family	n-hexane [class: Alkane], Toluene [class: Aromatic hydrocarbons], Dimethylphthalate [class: Pthalate]	olvent-resistant efflux pump outer membrane protein SrpC; The outer membrane component of an organic solvent efflux pump srpABC. Involved in export of a number of low log POW compounds including hexane (log POW 3.5), toluene (log POW 2.5) and dimethylphthalate (log POW 2.3). The solvent resistance phenotype has been postulated to depend on the operon expression level.	Kieboom et al. 1998; Pubmed-9417051, Kieboom eta l. 1998; Pubmed-9852029, Kim et al. 1998; Pubmed-9658016	efflux transporter outer membrane subunit	WP_024591829.1	L659_RS0121220
acrD	BAC0563	Efflux	RND superfamily, AcrB/AcrD/AcrF family	Copper (Cu), Zinc (Zn)	Acriflavine resistance protein D; participates in the efflux of aminoglycosides. It confers resistance to a variety of these substances. It contributes to copper and zinc resistance in Salmonella.	Nishino et al. 2007; Pubmed-17933888	efflux RND transporter permease subunit	WP_024591830.1	L659_RS0121225
mdtB	BAC0646	Efflux	RND superfamily, AcrB/AcrD/AcrF family	Zinc (Zn)	Multidrug resistance protein MdtB. Part of a tripartite efflux system composed of MdtA, MdtB and MdtC. MdtB forms a heteromultimer with MdtC. MdtABC multidrug efflux systems has a role in metal resistance.	Nishino et al. 2007; Pubmed-17933888	efflux RND transporter permease subunit	WP_024591830.1	L659_RS0121225
mdtC	BAC0647	Efflux	RND superfamily, AcrB/AcrD/AcrF family	Zinc (Zn)	Multidrug resistance protein MdtC. Part of a tripartite efflux system composed of MdtA, MdtB and MdtC. MdtB forms a heteromultimer with MdtC. MdtABC multidrug efflux systems has a role in metal resistance.	Nishino et al. 2007; Pubmed-17933888	efflux RND transporter permease subunit	WP_024591830.1	L659_RS0121225

mdtA	BAC0645	Efflux	RND superfamily, MFP family	Zinc (Zn)	Multidrug resistance protein MdtA. Part of a tripartite efflux system composed of MdtA, MdtB and MdtC. MdtB forms a heteromultimer with MdtC. MdtABC multidrug efflux systems has a role in metal resistance.	Nishino et al. 2007; Pubmed-17933888	efflux RND transporter periplasmic adaptor subunit	WP_024591831.1	L659_RS0121230
czcD	BAC0122	Efflux	Cation diffusion facilitator (CDF) transporter family	Cadmium (Cd), Zinc (Zn), Cobalt (Co)	Transporter gene influence Zn++ and Cd++ resistance; Necessary for activation of the czc determinant; may function as a membrane-bound sensor protein or as a metal ion transporter.	Nies et al. 1989; Pubmed-2678100	cation diffusion facilitator family transporter	WP_024591861.1	L659_RS0121430
zitB/ybgR	BAC0459	Efflux	Cation diffusion facilitator (CDF) transporter family	Zinc (Zn)	Zinc transporter ZitB; Involved in zinc efflux across the cytoplasmic membrane, thus reducing zinc accumulation in the cytoplasm and rendering bacteria more resistant to zinc; contributes to Zn++ haemostatis at low (non-toxic) concentrations; Appears to be selective for zinc, not conferring resistance to cobalt nor cadmium	Grass et al. 2001; Pubmed-11443104, Lee et al. 2002; Pubmed-12399046	cation diffusion facilitator family transporter	WP_024591861.1	L659_RS0121430
bfrA	BAC0048	Binding protein	Bacterioferritin family	Iron (Fe)	Bacterioferritin bfrA; Iron-storage protein, whose ferroxidase center binds Fe2+ ions, oxidizes them by dioxygen to Fe3+, and participates in the subsequent Fe3+ oxide mineral core formation within the central cavity of the protein complex	Gold et al. 2001; Pubmed-11722747	bacterioferritin	WP_010554007.1	L659_RS0121450
pbrR	BAC0301	Regulator	MerR family	Lead (Pb)	A transcription factor, pbrR belonging to the merR family	Borremans et al. 2001; Pubmed-11544228	Cd(II)/Pb(II)-responsive transcriptional regulato	WP_024592054.1	L659_RS0122630

zntR/yhdM	BAC0462	Regulator	Contains 1 HTH merR-type DNA-binding domain	Zinc (Zn)	Zinc-responsive transcriptional regulator zntR; regulates the zntA	Brocklehurst et al. 1999; Pubmed-10048032, Singh et al. 1999; Pubmed-10411736	Cd(II)/Pb(II)-responsive transcriptional regula	WP_024592054.1	L659_RS0122630
cznA	BAC0127	Efflux	RND superfamily, AcrB/AcrD/AcrF family	Cadmium (Cd), Zinc (Zn), Nickel (Ni)	Cadmium-zinc-nickel resistance protein cznA	Stahler et al. 2006; Pubmed-16790756	CusA/CzcA family heavy metal efflux RND transporter	WP_024592060.1	L659_RS0122660
nrsA	BAC0286	Membrane Transporter	RND superfamily, AcrB/AcrD/AcrF family	Nickel (Ni)	Cation or drug efflux system protein nrsA	Garcia-Dominquez et al. 2000; Pubmed-10692354	CusA/CzcA family heavy metal efflux RND transporter	WP_024592060.1	L659_RS0122660

Table S3. Genes putatively involved in antibiotic resistance identified in the *Pseudoalteromonas* sp. S8-8 genome

RGI criteria	ARO term	SNP	Detection criteria	AMR gene family	Drug class	Resistance mechanism	% Identity of Matching region	% Lenght of Reference sequence
Loose	ParR		protein homolog model	resistance-nodulation-cell division (RND) antibiotic efflux pump, Outer Membrane Porin (Opr)	macrolide antibiotic, fluoroquinolone antibiotic, monobactam, aminoglycoside antibiotic, carbapenem, cephalosporin, cephamycin, penam, tetracycline antibiotic, acridine dye, phenicol antibiotic, penem, disinfecting agents and intercalating dyes	antibiotic efflux, reduced permeability to antibiotic	33.33	99.57
Loose	evgS		protein homolog model	major facilitator superfamily (MFS) antibiotic efflux pump, resistance-nodulation-cell division (RND) antibiotic efflux pump	macrolide antibiotic, fluoroquinolone antibiotic, penam, tetracycline antibiotic	antibiotic efflux	36.23	33.67
Loose	novA		protein homolog model	ATP-binding cassette (ABC) antibiotic efflux pump	aminocoumarin antibiotic	antibiotic efflux	27.43	115.93
Loose	mtrA		protein homolog model	resistance-nodulation-cell division (RND) antibiotic efflux pump	macrolide antibiotic, penam	antibiotic efflux	41.88	322.37
Loose	evgS		protein homolog model	major facilitator superfamily (MFS) antibiotic efflux pump, resistance-nodulation-cell division (RND) antibiotic efflux pump	macrolide antibiotic, fluoroquinolone antibiotic, penam, tetracycline antibiotic	antibiotic efflux	25.44	89.56
Loose	FIM-1		protein homolog model	FIM beta-lactamase	carbapenem, cephalosporin	antibiotic inactivation	31.46	129.77
Loose	MuxA		protein homolog model	resistance-nodulation-cell division (RND) antibiotic efflux pump	macrolide antibiotic, monobactam, tetracycline antibiotic, aminocoumarin antibiotic	antibiotic efflux	25.55	121.36

Loose	adeB	protein homolog model	resistance-nodulation-cell division (RND) antibiotic efflux pump	glycylcycline, tetracycline antibiotic	antibiotic efflux	22.7	100.39
Loose	msbA	protein homolog model	ATP-binding cassette (ABC) antibiotic efflux pump	nitroimidazole antibiotic	antibiotic efflux	29.32	47.25
Loose	YajC	protein homolog model	resistance-nodulation-cell division (RND) antibiotic efflux pump	fluoroquinolone antibiotic, cephalosporin, glycylcycline, penam, tetracycline antibiotic, rifamycin antibiotic, phenicol antibiotic, triclosan	antibiotic efflux	45.22	99.11
Loose	PAC-1	protein homolog model	PAC beta-lactamase	cephalosporin	antibiotic inactivation	24.4	134.65
Loose	macB	protein homolog model	ATP-binding cassette (ABC) antibiotic efflux pump	macrolide antibiotic	antibiotic efflux	33.33	34.94
Loose	cpxA	protein homolog model	resistance-nodulation-cell division (RND) antibiotic efflux pump	aminoglycoside antibiotic, aminocoumarin antibiotic	antibiotic efflux	36.01	103.06
Loose	Pseudomonas aeruginosa CpxR	protein homolog model	resistance-nodulation-cell division (RND) antibiotic efflux pump	macrolide antibiotic, fluoroquinolone antibiotic, monobactam, aminoglycoside antibiotic, carbapenem, cephalosporin, cephamycin, penam, tetracycline antibiotic, peptide antibiotic, aminocoumarin antibiotic, diaminopyrimidine antibiotic, sulfonamide antibiotic, phenicol antibiotic, penem	antibiotic efflux	44.25	101.78
Loose	RanA	protein homolog model	ATP-binding cassette (ABC) antibiotic efflux pump	aminoglycoside antibiotic	antibiotic efflux	30.73	135.27
Loose	dfrA3	protein homolog model	trimethoprim resistant dihydrofolate reductase dfr	diaminopyrimidine antibiotic	antibiotic target replacement	55.56	100.62
Loose	macB	protein homolog model	ATP-binding cassette (ABC) antibiotic efflux pump	macrolide antibiotic	antibiotic efflux	34.83	36.02
Loose	evgS	protein homolog model	major facilitator superfamily (MFS) antibiotic efflux pump, resistance-nodulation-cell division (RND) antibiotic efflux pump	macrolide antibiotic, fluoroquinolone antibiotic, penam, tetracycline antibiotic	antibiotic efflux	28.75	106.02
Loose	vanRM	protein homolog model	glycopeptide resistance gene cluster, vanR	glycopeptide antibiotic	antibiotic target alteration	40.83	128.88
Loose	vanHD	protein homolog model	vanH, glycopeptide resistance gene cluster	glycopeptide antibiotic	antibiotic target alteration	29.82	97.21

Loose	TolC		protein homolog model	ATP-binding cassette (ABC) antibiotic efflux pump, major facilitator superfamily (MFS) antibiotic efflux pump, resistance-nodulation-cell division (RND) antibiotic efflux pump	macrolide antibiotic, fluoroquinolone antibiotic, aminoglycoside antibiotic, carbapenem, cephalosporin, glycylcycline, cephamycin, penam, tetracycline antibiotic, peptide antibiotic, aminocoumarin antibiotic, rifamycin antibiotic, phenicol antibiotic, triclosan, penem	antibiotic efflux	40.71	91.11
Loose	vanRE		protein homolog model	glycopeptide resistance gene cluster, vanR	glycopeptide antibiotic	antibiotic target alteration	26.95	173.80
Loose	NmcR		protein homolog model	NmcA beta-lactamase	carbapenem, cephalosporin, cephamycin, penam	antibiotic inactivation	42.42	98.31
Loose	lmrD		protein homolog model	ATP-binding cassette (ABC) antibiotic efflux pump	lincosamide antibiotic	antibiotic efflux	30.32	40.66
Loose	Clostridioides difficile gyrB conferring resistance to fluoroquinolones	V423F, I139R	protein variant model	fluoroquinolone resistant gyrB	fluoroquinolone antibiotic	antibiotic target alteration	39.02	98.74
